# Characterizing Associations and SNP-Environment Interactions for GWAS-Identified Prostate Cancer Risk Markers—Results from BPC3

**DOI:** 10.1371/journal.pone.0017142

**Published:** 2011-02-24

**Authors:** Sara Lindstrom, Fredrick Schumacher, Afshan Siddiq, Ruth C. Travis, Daniele Campa, Sonja I. Berndt, W. Ryan Diver, Gianluca Severi, Naomi Allen, Gerald Andriole, Bas Bueno-de-Mesquita, Stephen J. Chanock, David Crawford, J. Michael Gaziano, Graham G. Giles, Edward Giovannucci, Carolyn Guo, Christopher A. Haiman, Richard B. Hayes, Jytte Halkjaer, David J. Hunter, Mattias Johansson, Rudolf Kaaks, Laurence N. Kolonel, Carmen Navarro, Elio Riboli, Carlotta Sacerdote, Meir Stampfer, Daniel O. Stram, Michael J. Thun, Dimitrios Trichopoulos, Jarmo Virtamo, Stephanie J. Weinstein, Meredith Yeager, Brian Henderson, Jing Ma, Loic Le Marchand, Demetrius Albanes, Peter Kraft

**Affiliations:** 1 Program in Molecular and Genetic Epidemiology, Harvard School of Public Health, Boston, Massachusetts, United States of America; 2 Department of Epidemiology, Harvard School of Public Health, Boston, Massachusetts, United States of America; 3 Department of Preventive Medicine, Keck School of Medicine, University of Southern California, Los Angeles, California, United States of America; 4 Department of Epidemiology and Biostatistics, School of Public Health, Imperial College, London, United Kingdom; 5 Cancer Epidemiology Unit, Nuffield Department of Clinical Medicine, University of Oxford, Oxford, United Kingdom; 6 Genomic Epidemiology Group, German Cancer Research Center (DKFZ), Heidelberg, Germany; 7 Division of Cancer Epidemiology and Genetics, National Cancer Institute, National Institutes of Health, Bethesda, Maryland, United States of America; 8 Department of Epidemiology, American Cancer Society, Atlanta, Georgia, United States of America; 9 Cancer Epidemiology Centre, Cancer Council Victoria and the Centre for Molecular, Genetic, Environmental, and Analytic Epidemiology, University of Melbourne, Melbourne, Australia; 10 Division of Urologic Surgery, Washington University School of Medicine, St. Louis, Missouri, United States of America; 11 National Institute for Public Health and the Environment (RIVM), Bilthoven, The Netherlands; 12 Urologic Oncology, University of Colorado at Denver Health Sciences Center, Denver, Colorado, United States of America; 13 Massachusetts Veterans Epidemiology and Research Information Center (MAVERIC) and Geriatric Research, Education, and Clinical Center (GRECC), Boston Veterans Affairs Healthcare System, Boston, Massachusetts, United States of America; 14 Division of Aging, Department of Medicine, Brigham and Women's Hospital, Boston, Massachusetts, United States of America; 15 Department of Epidemiology and Preventive Medicine, Monash University, Melbourne, Australia; 16 Department of Nutrition, Harvard School of Public Health, Boston, Massachusetts, United States of America; 17 Division of Epidemiology, NYU Langone Medical Center, New York, New York, United States of America; 18 The Danish Cancer Society, Institute of Cancer Epidemiology, Copenhagen, Denmark; 19 Department of Medicine, Channing Laboratory, Brigham and Women's Hospital and Harvard Medical School, Boston, Massachusetts, United States of America; 20 International Agency for Research on Cancer, Lyon, France; 21 Department of Surgical and Perioperative Sciences, Urology and Andrology, Umeå University, Umeå, Sweden; 22 Division of Cancer Epidemiology, German Cancer Research Center (DKFZ), Heidelberg, Germany; 23 Epidemiology Program, Cancer Research Center, University of Hawaii, Honolulu, Hawaii, United States of America; 24 Department of Epidemiology, Regional Health Authority, Murcia, Spain; 25 CIBER Epidemiología y Salud Pública (CIBERESP), Barcelona, Spain; 26 CPO-Piemonte Torino, and Human Genetics Foundation, Torino, Italy; 27 Center for Food and Nutrition Policies, Athens, Greece; 28 Department of Chronic Disease Prevention, National Institute for Health and Welfare, Helsinki, Finland; 29 Department of Biostatistics, Harvard School of Public Health, Boston, Massachusetts, United States of America; Université de Montreal, Canada

## Abstract

Genome-wide association studies (GWAS) have identified multiple single nucleotide polymorphisms (SNPs) associated with prostate cancer risk. However, whether these associations can be consistently replicated, vary with disease aggressiveness (tumor stage and grade) and/or interact with non-genetic potential risk factors or other SNPs is unknown. We therefore genotyped 39 SNPs from regions identified by several prostate cancer GWAS in 10,501 prostate cancer cases and 10,831 controls from the NCI Breast and Prostate Cancer Cohort Consortium (BPC3). We replicated 36 out of 39 SNPs (P-values ranging from 0.01 to 10^−28^). Two SNPs located near *KLK3* associated with PSA levels showed differential association with Gleason grade (rs2735839, P = 0.0001 and rs266849, P = 0.0004; case-only test), where the alleles associated with decreasing PSA levels were inversely associated with low-grade (as defined by Gleason grade <8) tumors but positively associated with high-grade tumors. No other SNP showed differential associations according to disease stage or grade. We observed no effect modification by SNP for association with age at diagnosis, family history of prostate cancer, diabetes, BMI, height, smoking or alcohol intake. Moreover, we found no evidence of pair-wise SNP-SNP interactions. While these SNPs represent new independent risk factors for prostate cancer, we saw little evidence for effect modification by other SNPs or by the environmental factors examined.

## Introduction

Prostate cancer is the most common non-skin cancer among men in industrialized countries, but beyond age, ethnicity and family history, very little is known about its etiology. Observed familial aggregation together with evidence from both twin and epidemiological studies demonstrate a key role for inherited genetic variants [1].

Genome-wide association studies (GWAS) conducted within the last few years have identified multiple common single nucleotide polymorphisms (SNPs) associated with prostate cancer risk [2]–[15]. However, the function of these SNPs (or the causal variants these SNPs serve as proxies for) remains largely unknown and data describing their correlation with clinical factors or their interplay with other genetic and non-genetic factors are sparse, mainly due to the large sample sizes needed for sufficient statistical power.

To this end, we selected 39 SNPs from regions identified in previous GWAS and genotyped them in 10,501 prostate cancer cases and 10,831 controls within the NCI Breast and Prostate Cancer Cohort Consortium (BPC3). We tested each SNP for association with two strongly predictive clinical factors: Gleason grade and tumor stage. We investigated interactions between SNPs and known and potential environmental risk factors. Finally, we performed exploratory analysis to identify possible pair-wise SNP-SNP interactions.

## Results

### Association between SNPs and prostate cancer risk

Subject characteristics are displayed in Table 1. All 39 SNPs were significantly associated with prostate cancer risk (Table 2) and directions of associations were consistent with previous findings [2]–[4], [6]–[8], [10], [12], [14]. Although risk estimates varied somewhat between different cohorts (Table S1 and Figure S1), we observed overall no strong statistical evidence for heterogeneity (P>0.01). Risk effects per allele ranged from 1.06 (rs2928679) to 1.44 (rs16901979). Carriers of two copies of the rare ‘A’ allele of rs16901979 had a 3-fold increased risk to develop prostate cancer in this population. The allele frequency of rs16901979 varies widely across ethnicities (Hapmap population frequencies: 0.03 in CEU, 0.26 in CHB and 0.58 in YRI), and thus might explain a part of the differences seen in prostate cancer incidence across populations. Based on p-value, we observed the strongest association for rs4430796 located in *HNF1B/TCF2* (OR: 0.80 (95% CI: 0.77–0.83), P = 2.09•10^−28^) and the weakest association for rs4961199 near *CPNE3* (OR: 1.07 (95% CI: 1.02–1.14), P = 0.012). In addition, rs266849 near *KLK3* was only weakly associated with prostate cancer risk (OR: 0.93 (95% CI: 0.89–0.98), P = 0.009). rs266849 was initially identified in a GWAS using controls selected for low prostate-specific antigen (PSA) levels (<0.5 ng/ml) [4] and it has been suggested that rs266849 is a marker for circulating PSA levels rather than for prostate cancer risk [16], [17].

**Table 1:** Characteristics of the study populations.

	ATBC		CPS-II		EPIC		HPFS		MCCS		MEC		PHS		PLCO	
	Cases	Controls	Cases	Controls	Cases	Controls	Cases	Controls	Cases	Controls	Cases	Controls	Cases	Controls	Cases	Controls
**Number of Individuals**	1,036	952	2,285	2,286	1,581	1,845	1,288	1,252	1,031	1,397	668	743	1,381	1,303	1,231	1,053
**Mean** 1 **age (sd)**	69.6(5.7)	69.0(5.7)	70.1(5.7)	70.2(5.7)	65.4(6.3)	65.4(6.3)	69.6(7.5)	67.6(7.6)	67.6(6.9)	54.4(8.8)	69.0(7.5)	70.4(7.3)	70.3(7.6)	70.2(7.6)	67.0(5.5)	69.0(5.7)
**≤65 years (%)**	259(25)	263(28)	470(21)	484(21)	806(51)	946(51)	357(28)	458(37)	378(37)	1,211(87)	206(31)	199(27)	377(27)	369(28)	470(38)	289(27)
**>65 years (%)**	777(75)	689(72)	1,815(79)	1,802(79)	775(49)	899(49)	931(72)	794(63)	653(63)	186(13)	462(69)	544(73)	1,004(73)	934(72)	761(62)	764(73)
**FH + (%)**	56(3)	29(3)	494(22)	300(13)	NA	NA	201(16)	143(11)	118(11)	99(7)	76(11)	59(8)	NA	NA	143(12)	62(6)
**Missing (%)**	134(13)	114(12)	0 (0)	0 (0)	1,581(100)	1,845(100)	0 (0)	0(0)	313(30)	373(27)	37(6)	36(5)	1,303(100)	1,303(!00)	37(3)	16(2)
**BMI (%)**																
<25	395(38)	364(38)	893(40)	821(36)	517(33)	582(32)	524(47)	505(46)	272(26)	412(29)	244(37)	265(36)	798(58)	769(59)	327(27)	259(25)
25–30	504(49)	449(47)	1,118(50)	1,149(51)	846(54)	953(52)	497(44)	475(43)	581(56)	748(54)	328(49)	361(49)	541(39)	490(38)	631(52)	535(51)
>30	137(13)	138(15)	247(10)	285(13)	218(14)	310(17)	100(9)	116(11)	178(17)	237(17)	96(14)	116(16)	42(3)	44(3)	258(21)	249(24)
**Missing (%)**	0(0)	1(0)	27(1)	31(1)	0(0)	0(0)	167(13)	156(12)	0(0)	0(0)	0(0)	1(0)	0(0)	0(0)	15(1)	10(1)
**Height (%)**																
**<173 cm**	493(52)	486(47)	532(22)	542(25)	826(49)	808(46)	293(28)	322(25)	678(56)	606(50)	238(35)	211(29)	275(22)	338(24)	283(25)	290(26)
**173**–**180** **cm**	342(36)	405(39)	633(26)	652(30)	613(36)	646(37)	387(37)	632(49)	399(33)	399(33)	167(25)	216(29)	504(40)	651(46)	346(30)	332(29)
**>180 cm**	120(12)	141(14)	1,227(52)	952(45)	246(15)	287(17)	371(35)	348(24)	141(11)	205(17)	269(40)	309(42)	475(38)	441(30)	510(45)	506(45)
**Missing (%)**	0(0)	1(0)	21(1)	12(1)	0(0)	0(0)	101(8)	86(7)	0(0)	0(0)	0(0)	1(0)	0(0)	0(0)	11(1)	6(1)
**Smoking (%)**																
Never	0(0)	0(0)	816 (36)	838 (37)	547 (35)	622 (34)	605(48)	532(44)	444(43)	608(44)	233(35)	249(34)	671(49)	658(50)	507(41)	395(38)
Former	0(0)	0(0)	661 (58)	1,330 (59)	661(42)	753(41)	593(47)	638(52)	499(48)	594(43)	364(55)	406(55)	589(43)	544(42)	627(51)	541(51)
Current	1,036(100)	952(100)	348(6)	105(5)	348(22)	443(24)	56(4)	49(4)	88(9)	195(14)	66(10)	84(11)	119(9)	101(8)	96(8)	117(11)
**Missing (%)**	0(0)	0(0)	17(1)	13(1)	25(2)	27(1)	34(3)	33(3)	0(0)	0(0)	5(1)	4(1)	2(0)	0(0)	1(0)	0(0)
**Alcohol Consumption (%)**																
<30 g/day	816(79)	740(78)	1,867(82)	1,890(84)	1,206(76)	1,443(78)	1,106(86)	1,070(85)	757(73)	1,030(74)	484(70)	560(75)	1,375(100)	1,293(99)	945(77)	816(77)
≥30 g/day	176(17)	165(17)	291(13)	247(11)	375(24)	402(22)	143(11)	135(11)	272(26)	367(26)	162(24)	152(20)	0(0)	0(0)	203(16)	167(16)
**Missing (%)**	44(4)	47 (5)	127 (5)	149 (5)	0(0)	0(0)	39 (3)	47(4)	2(1)	0(0)	22(6)	31(5)	6(0)	10(1)	83(7)	70(7)
**Diabetes (%)**	34 (3)	24 (3)	100 (4)	150 (7)	66 (5)	95 (6)	67 (5)	59 (5)	34 (3)	51 (4)	25 (4)	38 (5)	21 (2)	19 (1)	82 (7)	117 (11)
**Missing (%)**	0(0)	0(0)	0(0)	0(0)	279(18)	357(18)	0(0)	0(0)	0(0)	0(0)	0(0)	0(0)	2(0)	1(0)	34(3)	34(3)
**High Grade** 2 **(%)**	199(19)	NA	323(14)	NA	75(5)	NA	114(9)	NA	148(14)	NA	206(30)	NA	197(14)	NA	126(10)	NA
**Missing (%)**	174(!7)	NA	326(1$)	NA	780(49)	NA	190(15)	NA	27(3)	NA	28(4)	NA	38(3)	NA	2(0)	NA
**High Stage** 3 **(%)**	258(25)	NA	409(18)	NA	257(16)	NA	139(11)	NA	93(9)	NA	85(11)	NA	231(17)	NA	245(20)	NA
**Missing (%)**	205(20)	NA	65(3)	NA	506(32)	NA	197(15)	NA	34(3)	NA	15(2)	NA	572(41)	NA	0(0)	NA

1 Age is calculated at age of diagnosis/selection as control except for MCCS (at baseline for controls) and MEC (at blood draw for controls).

2 Defined as Gleason 8–10 or WHO grade III.

3 Defined as Stage CD.

**Table 2:** Associations between selected SNPs and prostate cancer risk.

SNP	Pos	Chr	Gene	N Cases	N Controls	MAF Cases	MAF Controls	OR Het	OR Hom	OR allele	P-value	Ref
rs721048	62,985,235	2	*EHBP1*	10160	10474	0.19	0.18	1.12 (1.06–1.19)	1.22 (1.04–1.42)	1.11 (1.06–1.17)	2.47E-05	1
rs1465618	43,407,453	2	*THADA*	9758	9627	0.23	0.21	1.09 (1.00–1.19)	1.18 (0.97–1.43)	1.12 (1.06–1.17)	1.02E-05	12
rs12621278	173,019,799	2	*ITGA6*	9822	9667	0.05	0.06	0.88 (0.76–1.02)	0.32 (0.13–0.83)	0.86 (0.78–0.94)	0.0010	12
rs2660753	87,193,364	3	*–*	10000	10502	0.12	0.11	1.12 (1.05–1.20)	1.32 (1.05–1.66)	1.13 (1.06–1.20)	9.18E-05	2
rs4857841	129,529,333	3	*EEFSEC*	9589	10103	0.30	0.28	1.13 (1.07–1.20)	1.28 (1.15–1.42)	1.13 (1.08–1.18)	2.32E-08	3
rs17021918	95,781,900	4	*PDLIM5*	9791	9702	0.32	0.34	0.88 (0.81–0.96)	0.84 (0.73–0.97)	0.92 (0.88–0.96)	5.35E-05	12
rs12500426	95,733,632	4	*PDLIM5*	9784	9643	0.48	0.47	1.07 (0.97–1.19)	1.14 (1.02–1.29)	1.07 (1.02–1.11)	0.0021	12
rs7679673	106,280,983	4	*TET2*	9772	9613	0.39	0.42	0.82 (0.74–0.89)	0.74 (0.65–0.85)	0.88 (0.84–0.92)	6.74E-10	12
rs9364554	160,753,654	6	*SLC22A3*	9994	10464	0.29	0.28	1.08 (1.02–1.15)	1.15 (1.04–1.27)	1.08 (1.03–1.12)	0.00086	2
rs10486567	27,943,088	7	*JAZF1*	10242	10618	0.21	0.24	0.82 (0.78–0.87)	0.73 (0.64–0.83)	0.84 (0.80–0.88)	7.05E-14	4
rs6465657	97,654,263	7	*LMTK2*	9994	10465	0.48	0.46	1.13 (1.06–1.21)	1.23 (1.14–1.33)	1.11 (1.07–1.16)	1.52E-07	2
rs1512268	23,582,408	8	*NKX3*–*1*	9849	9732	0.46	0.44	1.12 (1.02–1.23)	1.28 (1.14–1.44)	1.11 (1.06–1.15)	5.52E-07	12
rs2928679	23,494,920	8	*SLC25A37*	9845	9697	0.46	0.44	1.01 (0.91–1.11)	1.16 (1.03–1.30)	1.06 (1.02–1.11)	0.0036	12
rs4961199	87,650,060	8	*CPNE3*,*CNGB3*	9184	9147	0.17	0.16	1.03 (0.97–1.10)	1.36 (1.13–1.64)	1.07 (1.02–1.14)	0.012	4
rs1016343	128,162,479	8	*–*	9636	10116	0.24	0.20	1.22 (1.15–1.30)	1.64 (1.44–1.88)	1.25 (1.19–1.31)	1.54E-19	2
rs7841060	128,165,659	8	*–*	9341	9859	0.24	0.20	1.22 (1.15–1.30)	1.63 (1.42–1.86)	1.24 (1.18–1.31)	1.43E-18	3
rs16901979	128,194,098	8	*–*	9120	9079	0.05	0.03	1.41 (1.26–1.58)	3.02 (1.47–6.23)	1.44 (1.29–1.60)	1.55E-11	5
rs620861	128,404,855	8	*–*	9097	9611	0.34	0.37	0.86 (0.81–0.92)	0.75 (0.68–0.82)	0.87 (0.83–0.90)	3.59E-11	3
rs6983267	128,482,487	8	*–*	10186	10521	0.44	0.49	0.82 (0.77–0.87)	0.66 (0.61–0.71)	0.81 (0.78–0.84)	6.62E-26	4
rs1447295	128,554,220	8	*–*	9640	10098	0.14	0.10	1.38 (1.29–1.48)	1.94 (1.53–2.47)	1.38 (1.30–1.47)	8.06E-25	6
rs4242382	128,586,755	8	*–*	10296	10630	0.14	0.10	1.41 (1.31–1.51)	1.95 (1.55–2.47)	1.40 (1.32–1.49)	4.17E-28	4
rs7837688	128,608,542	8	*–*	9624	10133	0.13	0.10	1.34 (1.25–1.44)	1.96 (1.54–2.50)	1.36 (1.27–1.44)	6.70E-22	7
rs16902094	128,389,528	8		8407	8378	0.18	0.16	1.23 (1.11–1.36)	1.10 (0.81–1.48)	1.18 (1.11–1.25)	4.78E-08	13
rs1571801	123,467,194	9	*–*	9961	9833	0.27	0.25	1.08 (1.02–1.15)	1.11 (0.99–1.24)	1.07 (1.02–1.12)	0.0049	8
rs10993994	51,219,502	10	*MSMB*	9982	10493	0.44	0.39	1.20 (1.12–1.27)	1.53 (1.41–1.66)	1.23 (1.18–1.28)	5.95E-25	4
rs4962416	126,686,862	10	*CTBP2*	10020	10348	0.29	0.27	1.07 (1.01–1.13)	1.23 (1.11–1.37)	1.09 (1.05–1.14)	7.92E-05	4
rs7127900	2190150	11		9793	9694	0.22	0.20	1.20 (1.09–1.31)	1.62 (1.31–2.00)	1.14 (1.09–1.20)	2.01E-07	12
rs12418451	68,691,995	11	*–*	9641	10129	0.31	0.29	1.12 (1.05–1.19)	1.27 (1.15–1.41)	1.12 (1.08–1.17)	1.42E-07	9
rs7931342	68,751,073	11	*–*	9765	10226	0.45	0.49	0.86 (0.81–0.92)	0.71 (0.66–0.77)	0.85 (0.81–0.88)	1.11E-16	2
rs10896449	68,751,243	11	*–*	10122	10504	0.45	0.50	0.86 (0.80–0.91)	0.70 (0.65–0.76)	0.84 (0.81–0.87)	3.58E-19	4
rs11649743	33,149,092	17	*TCF2*	10184	10538	0.17	0.19	0.87 (0.82–0.92)	0.76 (0.65–0.89)	0.87 (0.83–0.92)	7.55E-08	10
rs4430796	33,172,153	17	*TCF2*	9984	9785	0.44	0.49	0.80 (0.75–0.85)	0.64 (0.59–0.69)	0.80 (0.77–0.83)	2.09E-28	11
rs7501939	33,175,269	17	*TCF2*	9557	10082	0.36	0.40	0.80 (0.76–0.86)	0.69 (0.63–0.75)	0.82 (0.79–0.86)	2.78E-20	2
rs1859962	66,620,348	17	*–*	10202	10547	0.53	0.48	1.23 (1.15–1.32)	1.41 (1.31–1.53)	1.19 (1.14–1.24)	2.74E-18	11
rs266849	56,040,902	19	*KLK3*	10015	10348	0.18	0.19	0.91 (0.86–0.97)	0.94 (0.81–1.09)	0.93 (0.89–0.98)	0.0085	2
rs2735839	56,056,435	19	*KLK3*	9862	10366	0.13	0.15	0.90 (0.84–0.96)	0.67 (0.54–0.82)	0.87 (0.82–0.92)	3.05E-06	2
rs5759167	41,830,156	22	*BIK*	9843	9729	0.47	0.50	0.83 (0.75–0.91)	0.70 (0.62–0.79)	0.87 (0.83–0.90)	1.30E-12	12
rs5945572	51,246,423	X	*NUDT11*	9338	9830	0.40	0.35			1.22 (1.15–1.29)	6.17E-11	1
rs5945619	51,258,412	X	*NUDT11*	9999	10456	0.41	0.36			1.23 (1.16–1.31)	8.27E-13	2

1.Gudmundsson, et al. 2008.

2.Eeles, et al. 2008.

3.Yeager, et al. 2009.

4.Thomas, et al. 2008.

5.Gudmundsson, et al. 2007.

6.Amundadottir, et al. 2006.

7.Yeager, et al. 2007.

8.Duggan, et al. 2007.

9.Zheng, et al. 2009.

10.Sun, et al. 2009.

11.Gudmundsson, et al. 2007.

12. Eeles et al. 2009

13.Gudmundsson et al 2009

The primary analysis in most GWAS assumes an additive increase in risk for each risk allele carried. rs4961199 (P = 0.02) was the only SNP showing nominally significant evidence of departure from additivity in our data. This was not unexpected since rs4961199 was initially identified using a recessive inheritance model [12].

### Replication in non-CGEMS cohorts

Eleven of the 39 SNPs included in this paper were identified through the Cancer Genetic Markers of Susceptibility (CGEMS) project (http://cgems.cancer.gov/) which has partial overlap with BPC3. Eight of these eleven SNPs were identified through CGEMS stage 2 including ATBC, CPS-II, HPFS and PLCO. We attempted to replicate these SNPs in the other cohorts (EPIC, MCCS, MEC and PHS) which collectively comprise 4,661 prostate cancer cases and 5,288 controls. Out of these eight SNPs, six replicated (Table S2) and two did not (rs4961199 near *CPNE3* (OR: 0.99 (95% CI: 0.91–1.08), P = 0.82) and rs4962416 in *CTBP2* (OR: 1.01 (95% CI: 0.95–1.08), P = 0.73)). Three SNPs (rs4857841 (*EFFSEC*), rs7841060 (8q24) and rs620861 (8q24)) were identified in CGEMS stage 3 (that in addition to ATBC, CPS-II, HPFS and PLCO included EPIC and MEC). Therefore, we tested these three SNPs in MCCS and PHS only (2,700 cases and 2,412 controls). Both rs7841060 (OR: 1.25 (95% CI: 1.12–1.40), P = 8.8•10^−5^) and rs620861 (OR: 0.89 (95% CI: 0.80–0.98), P = 0.01) were associated with prostate cancer risk whereas rs4857841 was not (OR: 1.03 (95% CI: 0.93–1.15), P = 0.52). Since we did not replicate rs4857841, rs4961199 and rs4962416 in the non-CGEMS studies, we did not pursue these SNPs in further analysis

### SNP associations by tumor stage and grade

We next examined whether any SNP was differentially associated with tumor grade or stage at diagnosis (Table 3). A total of 1,717 cases were classified as high-stage (stage C or D at diagnosis) and 1,388 were classified as high-grade (Gleason grade 8–10 or equivalent, i.e. coded as poorly differentiated or undifferentiated). For 15% of the cases, we did not have information about tumor stage or Gleason grade. The minor alleles of two SNPs in the *KLK3* gene (rs266849 and rs2735839), which have been previously associated with decreasing PSA levels [16], [17], were inversely associated with low-grade disease (Gleason <8) (OR: 0.91 (0.86–0.96) for rs266489, OR: 0.84 (0.79–0.89) for rs2735839) but associated with increased risk for high-grade disease (OR: 1.10 (1.00–1.22) for rs266489, OR: 1.07 (0.95–1.19) for rs2735839). The differences in SNP associations between low-grade and high-grade disease were statistically significant in case-only analysis (P = 0.0004 for rs266849 and P = 0.0001 for rs2735839). These results remained significant after Bonferroni correction (P = 0.014 for rs266849 and P = 0.0036 for rs2735839). No other SNP was differentially associated with tumor grade or stage after adjusting for multiple testing.

**Table 3:** Associations between selected SNPs and Gleason grade and Stage.

	Odds Ratio^1^ (95% CI)			Odds Ratio^1^ (95% CI)		
SNP	Gleason <8	Gleason 8–10	P case-only test	Stage AB	StageCD	P case-only test
rs721048	1.13 (1.07–1.20)	1.07 (0.96–1.19)	0.30	1.13 (1.07–1.20)	1.09 (0.99–1.19)	0.5
rs1465618	1.10 (1.05–1.16)	1.17 (1.06–1.30)	0.15	1.13 (1.07–1.19)	1.07 (0.97–1.17)	0.34
rs12621278	0.87 (0.79–0.96)	0.77 (0.62–0.95)	0.27	0.87 (0.78–0.96)	0.85 (0.71–1.02)	0.86
rs2660753	1.10 (1.03–1.18)	1.11 (0.98–1.26)	0.81	1.13 (1.05–1.21)	1.14 (1.01–1.27)	0.82
rs4857841	1.13 (1.07–1.18)	1.15 (1.05–1.26)	0.54	1.14 (1.09–1.20)	1.15 (1.06–1.25)	0.53
rs17021918	0.91 (0.87–0.95)	0.96 (0.88–1.05)	0.22	0.89 (0.58–0.93)	1.01 (0.93–1.09)	0.004
rs12500426	1.07 (1.03–1.12)	0.99 (0.91–1.08)	0.09	1.08 (1.04–1.13)	0.97 (0.89–1.04)	0.007
rs7679673	0.88 (0.84–0.92)	0.86 (0.79–0.94)	0.63	0.88 (0.84–0.92)	0.85 (0.78–0.92)	0.49
rs9364554	1.10 (1.05–1.15)	1.03 (0.94–1.13)	0.28	1.07 (1.02–1.12)	1.13 (1.04–1.22)	0.30
rs10486567	0.83 (0.78–0.88)	0.93 (0.83–1.05)	0.06	0.81 (0.77–0.85)	0.91 (0.84–1.00)	0.02
rs6465657	1.10 (1.05–1.15)	1.09 (1.01–1.18)	0.80	1.12 (1.07–1.17)	1.03 (0.96–1.11)	0.02
rs1512268	1.11 (1.06–1.16)	1.02 (0.94–1.11)	0.08	1.12 (1.07–1.17)	1.05 (0.97–1.13)	0.05
rs2928679	1.07 (1.03–1.12)	1.09 (1.00–1.19)	0.81	1.08 (1.03–1.12)	1.05 (0.97–1.14)	0.78
rs4961199	1.10 (1.04–1.17)	1.08 (0.97–1.22)	0.82	1.08 (1.01–1.15)	1.10 (0.99–1.22)	0.70
rs1016343	1.24 (1.17–1.31)	1.31 (1.18–1.45)	0.33	1.26 (1.19–1.33)	1.25 (1.15–1.37)	0.92
rs7841060	1.23 (1.17–1.30)	1.31 (1.18–1.45)	0.32	1.26 (1.19–1.33)	1.24 (1.13–1.36)	0.82
rs16901979	1.43 (1.27–1.60)	1.39 (1.12–1.72)	0.91	1.40 (1.24–1.58)	1.55 (1.29–1.86)	0.33
rs620861	0.85 (0.81–0.89)	0.89 (0.81–0.97)	0.34	0.87 (0.83–0.91)	0.82 (0.75–0.89)	0.37
rs6983267	0.80 (0.77–0.84)	0.84 (0.77–0.91)	0.62	0.82 (0.78–0.85)	0.82 (0.76–0.89)	0.54
rs1447295	1.41 (1.32–1.51)	1.31 (1.16–1.49)	0.35	1.36 (1.27–1.46)	1.51 (1.36–1.68)	0.06
rs4242382	1.43 (1.34–1.53)	1.38 (1.22–1.55)	0.73	1.39 (1.30–1.49)	1.49 (1.34–1.66)	0.24
rs7837688	1.39 (1.30–1.49)	1.33 (1.18–1.51)	0.72	1.34 (1.25–1.44)	1.47 (1.32–1.64)	0.09
rs16902094	1.19 (1.12–1.27)	1.07 (0.94–1.21)	0.08	1.18 (1.10–1.26)	1.18 (1.06–1.31)	0.74
rs1571801	1.07 (1.02–1.13)	1.10 (1.01–1.21)	0.50	1.07 (1.02–1.13)	1.09 (1.00–1.19)	0.62
rs10993994	1.23 (1.18–1.29)	1.21 (1.12–1.32)	0.94	1.25 (1.19–1.30)	1.20 (1.11–1.29)	0.29
rs4962416	1.10 (1.05–1.16)	1.09 (1.00–1.20)	0.93	1.10 (1.05–1.15)	1.10 (1.01–1.19)	0.88
rs7127900	1.14 (1.08–1.21)	1.05 (0.95–1.17)	0.14	1.14 (1.08–1.20)	1.16 (1.06–1.28)	0.81
rs12418451	1.11 (1.06–1.17)	1.20 (1.10–1.31)	0.09	1.14 (1.08–1.19)	1.06 (0.97–1.15)	0.14
rs7931342	0.86 (0.82–0.90)	0.81 (0.75–0.88)	0.21	0.85 (0.81–0.89)	0.88 (0.81–0.94)	0.33
rs10896449	0.85 (0.82–0.89)	0.80 (0.74–0.87)	0.13	0.84 (0.80–0.87)	0.86 (0.80–0.93)	0.34
rs11649743	0.88 (0.83–0.93)	0.93 (0.84–1.03)	0.40	0.89 (0.84–0.94)	0.84 (0.76–0.93)	0.21
rs4430796	0.78 (0.75–0.81)	0.84 (0.77–0.91)	0.05	0.81 (0.77–0.84)	0.78 (0.73–0.85)	0.47
rs7501939	0.81 (0.77–0.85)	0.85 (0.77–0.92)	0.30	0.83 (0.80–0.87)	0.80 (0.74–0.86)	0.28
rs1859962	1.20 (1.15–1.25)	1.17 (1.08–1.27)	0.70	1.18 (1.13–1.24)	1.22 (1.13–1.31)	0.58
rs266849	0.91 (0.86–0.96)	1.10 (1.00–1.22)	0.0004	0.92 (0.87–0.97)	0.98 (0.89–1.08)	0.18
rs2735839	0.84 (0.79–0.89)	1.07 (0.95–1.19)	0.0001	0.85 (0.80–0.91)	0.92 (0.83–1.03)	0.17
rs5759167	0.87 (0.83–0.91)	0.85 (0.78–0.93)	0.59	0.88 (0.84–0.92)	0.82 (0.76–0.89)	0.11
rs5945572	1.24 (1.16–1.32)	1.20 (1.06–1.36)	0.56	1.22 (1.14–1.31)	1.27 (1.14–1.42)	0.57
rs5945619	1.26 (1.19–1.34)	1.19 (1.05–1.34)	0.26	1.22 (1.14–1.30)	1.33 (1.20–1.28)	0.16

1 Odds ratios were estimated using multinomial regression.

### Association between non-genetic factors and prostate cancer risk

We tested for association between prostate cancer risk and potential non-genetic risk factors including family history of prostate cancer, diabetes, BMI, height, smoking and alcohol consumption. As expected, we observed a strong association between family history of prostate cancer and prostate cancer risk (OR: 1.77, 95% CI: 1.59–1.96, P = 1.88•10^−27^) as well as between diabetes and prostate cancer risk (OR: 0.73, 95% CI: 0.64–0.83, P = 1.61•10^−6^). Adjusting for BMI did not alter the association between diabetes and prostate cancer (data not shown). BMI was inversely associated with prostate cancer risk (OR: 0.996 (95% CI: 0.994–0.998) per BMI unit increase, P = 0.0004). This association was limited to obese men (BMI >30) compared to normal weight men (BMI<25) (OR: 0.86, 95% CI: 0.79–0.94, P = 0.0009), and we observed no association for being overweight (OR: 0.99, 95% CI: 0.93–1.05, P = 0.64). Adjusting for diabetes and smoking attenuated the association between obesity and prostate cancer risk (OR: 0.89, 95% CI: 0.82–0.98, P = 0.02). The inverse association between BMI and prostate cancer risk was restricted to non-aggressive cases as defined by Gleason grade <8 and tumor stages A and B (data not shown). Height was not associated with prostate cancer risk, when analyzed as a continuous variable (OR: 1.001, 95% CI: 1.000–1.002 per cm increase, P = 0.12) or in tertiles (OR: 1.02, 95% CI: 0.99–1.06, P = 0.24). We observed a non-significant reduced prostate cancer risk among both former smokers (OR: 0.95, 95% CI: 0.89–1.01, P = 0.08) and current smokers (OR: 0.91, 95% CI: 0.82–1.00, P = 0.06) compared to never smokers. Adjusting for alcohol consumption or BMI did not change the results (data not shown). Finally, consuming more than 30 g alcohol per day (corresponding to two drinks) was associated with an increased prostate cancer risk (OR: 1.09, 95% CI: 1.01–1.18, P = 0.03). Adjusting for smoking did not alter this association (data not shown).

### SNP-environment and SNP-SNP interactions

To investigate if the associations with family history of prostate cancer, diabetes and BMI were stronger in specific genetic strata, we tested for effect modification by including a SNPxE interaction term in the model. We also tested for SNP effect modification of age at diagnosis (studying the main effect of age is not appropriate since our population comprises of a series of nested case-control studies matched on age). After adjusting for multiple testing, no SNP showed significant statistical interaction with any of the non-genetic factors tested (Table S3 and Table S4). Of note, two SNPs in the 8q24 region (rs620861, P = 0.05 and rs6983267, P = 0.004) showed nominally significant interactions with age at diagnosis, with the association being stronger in younger men. These results are in line with previous reports of stronger associations with earlier onset of disease for SNPs in the 8q24 region [6], [18], [19]. We observed marginally significant interactions between diabetes and rs10486567 in *JAZF1* (P = 0.04) and between BMI and rs10486567 (P = 0.03). This is of particular interest since genetic variation in *JAFF1* has been associated with diabetes, albeit not the same genetic variants. In this study, obesity was associated with a reduced risk for prostate cancer. It has been shown that BMI is inversely associated with PSA levels [20] and thus, obese men are less likely to get diagnosed through PSA screening. Because BMI was associated with non-aggressive disease, we also looked at possible SNP-BMI interactions stratified by disease aggressiveness but observed no significant interactions (data not shown).

To assess if the ambiguous associations between prostate cancer risk and height, smoking and alcohol consumption are due to hidden SNP-environment interactions, we conducted a joint test of the environmental main effect and the SNP-environment interaction effect. This test has proven powerful when the non-genetic effect is limited to a specific genetic stratum [21]. Across SNPs, the joint test was not significant for either alcohol or smoking after adjustment for multiple testing (Table S5, Table S6 and Table S7). Similarly, standard interaction tests between SNPs and height, SNPs and smoking and SNPs and alcohol consumption were not significant. Exploratory analyses of all possible pair-wise SNP-SNP interactions revealed no excess in significant interactions than expected by chance (50 out of 630 tests, Table S8). Furthermore, no SNP-SNP interaction was significant after correcting for multiple testing using a Bonferroni correction (lowest nominal P-value was 0.0005). Yeager and colleagues identified a SNP-SNP interaction between rs4242382 and rs620861 (P = 0.002) [13]. We also observe this interaction (P = 0.02), but not when the analysis was restricted to only MCCS and PHS (P = 0.75).

## Discussion

In this study, we set out to examine whether SNPs identified in GWAS to be associated with prostate cancer show variation in risk by disease aggressiveness (tumor stage and grade) and/or interact with non-genetic and genetic factors. All 39 SNPs tested were significantly associated with prostate cancer in the overall analysis. However, the CGEMS project, which included four and six BPC3 studies in its second and third stage stages, respectively, contributed to identification of eleven SNPs investigated in the present study. We tested whether associations for these eleven SNPs could be confirmed in the remaining studies and with the exception of three SNPs, the findings were replicated with risk magnitudes similar to those in the CGEMS analysis. We could not replicate rs4961149 using data from three of the non-CGEMS cohorts. Since rs4961199 was included in CGEMS stage 2 based on its recessive association, we also tested the recessive model in the non-CGEMS studies and observed a non-significant association similar but weaker as compared with CGEMS (OR: 1.10, 95% CI: 0.82–1.47, P = 0.54).

Few of the observed associations differed by disease stage, tumor grade or environmental exposures. The most noteworthy finding was the qualitatively altered association according to Gleason grade for two SNPs near *KLK3* (rs266849 and rs2735839), where the minor alleles were associated with lower risk of low-grade disease but higher risk of Gleason 8–10 tumors. This was previously observed by Kader and colleagues [22] who studied 5,000 patients and found a strong association between Gleason grade and rs2735839 (P = 3.7•10^−7^). The minor alleles of these SNPs have been associated with lower PSA levels indicating that carriers are less likely to be diagnosed at an early stage through PSA screening [16], [17]. However, we did not observe any difference in the association of these two SNPs by disease stage, suggesting that delayed diagnosis might not fully explain these associations. Interestingly, the significant positive association of these two SNPs with Gleason 8–10 tumors support the clinical observations that PSA expression is lower in malignant than in normal prostatic epithelium and is further reduced in poorly differentiated tumors [23], [24]. Together, these results suggest that *KLK* variation might influence high-grade prostate cancer risk through a yet unidentified pathway or simply as a genetic marker of the probability of a diagnosis of high versus low-grade prostate cancer diagnosis through its influence on PSA levels. To test this hypothesis, we performed case-only analysis based on year of diagnosis to reflect the introduction of wide-spread PSA screening (up to 1992 (670 men) vs. after 1992 (9831 men)). If the association between Gleason grade and *KLK3* variation is due to altered PSA levels, we would expect to see differential associations according to year of diagnosis. We did not observe such differences, however, suggesting that the *KLK3*-prostate cancer association is not mediated by altered PSA levels. A recent Icelandic study conducted stratified analysis based on year of diagnosis and noticed that the association with prostate cancer was confined to the group of cases diagnosed in 1992 or later. These results suggest that the association between the KLK3 locus and prostate cancer is driven by the increasing frequency of PSA testing [25].

After adjusting for multiple testing, no other SNP was associated with clinical sub-types. Earlier studies had failed to link these SNPs to clinical characteristics [22], [26], suggesting that these SNPs affect prostate cancer risk overall and not solely for more (or less) aggressive or advanced cancer.

We found overall no evidence that these SNPs interact with known or proposed risk factors for prostate cancer including family history of prostate cancer risk, age of onset, diabetes, BMI, height, smoking or alcohol consumption. Studying the interactions between SNPs and diabetes was of particular interest since genetic variation in *JAZF1* and *TCF2* has been associated with both prostate cancer and diabetes [8], [11], [12], [27], [28]. We did see a borderline statistically significant interaction between rs10486567 in *JAZF1* and diabetes, but this particular SNP has not been associated with diabetes risk. A previous study conducted in CPS-II and PLCO found that diabetes did not mediate the association between *JAZF1* and *HNF1B/TCF2* SNPs and prostate cancer risk [29], and we observed no statistical interaction between diabetes and three SNPs in *HNFIB/TCF2*.

We observed no significant associations between prostate cancer risk and smoking or height and only a weak association between prostate cancer and alcohol consumption, even after accounting for the possibility of differences in the effects of these exposures by genotype. A meta-analysis of 39 studies observed that height was positively associated with risk (RR 1.05 per 10 cm increment, 95% CI 1.02–1.09) but the association was only seen in cohort studies [30]. A recent large meta-analysis of smoking and prostate cancer incidence found overall no evidence of an association but reported an increased risk when considering number of cigarettes smoked. Moreover, they observed a 9% risk increase for former smokers [31]. We did observe a marginal association between alcohol intake and prostate cancer risk. This is in line with earlier results indicating a weak risk increase for men consuming at least 25 grams alcohol per day (OR: 1.05 (95% CI: 1.00–1.08)) and for men consuming at least 50 grams per day (OR: 1.09 (95% CI: 1.02–1.17)) [32].

Overall, these results imply that the lack of robust associations between these environmental factors and prostate cancer risk is not due to interactions between these exposures and variation in any of the 36 SNPs assessed in this study. However, the lack of significant interactions does not rule out that gene-environment interactions exist in prostate cancer. All SNPs under study have been linked to prostate cancer through their main effects. Agnostic approaches such as incorporating gene-environment interactions in a genome-wide association study setting might identify genetic variants that only affect risk when acting with other factors. The lack of significant interactions can also reflect the low power to detect only modest interaction effects despite our sample size of 10,000 cases and 10,000 controls. It is important to note that our results do not rule out small departures from a multiplicative odds model for the joint effect of pairs of individual markers and risk factors, nor does absence of departure from a multiplicative odds model necessarily imply that these genetic loci and risk factors do not interact in some causal manner. Moreover, absence of interaction as defined here does not imply absence of a “public health interaction”, where the benefit from reducing a risk factor in terms of absolute risk reduction differs across genotypes [33].

This is, to our knowledge, the first large-scale study to explore possible interactions between confirmed prostate cancer susceptibility markers and a broad spectrum of known and possible environmental factors. The SNPs considered in this study show marginal per-allele odds ratios ranging between 1.07 and 1.44. It is possible that these odds ratios might be larger in strata defined by other prostate cancer risk factors, not evaluated in this study. It is well recognized that exploring such interactions requires large study populations with well-defined exposure data. With 10,501 prostate cancer cases, 10,831 controls and prospectively collected data within established cohorts, BPC3 is in a unique position to explore both gene-gene and gene-environment interactions as demonstrated here. For example, in the absence of main effects (which is not the same as assuming no marginal effect and plausibly consistent with modest marginal genetic or environmental effects), the BPC3 has 89% power to detect an interaction effect of 1.2 assuming an allele frequency of 20% and an environmental exposure with a prevalence of 20%.

As with all studies utilizing environmental exposure data, the present investigation would be expected to have some degree of misclassification in the measurement of those factors. It is possible that alternative modeling of the environmental risk factors or more precise exposure quantification would increase statistical power (e.g. analyzing intensity, duration or pack-years of smoking rather than as never/former/current). However, a critical issue in conducting pooled analysis across studies is to harmonize data. As exposure data gets more refined, there is an increasing risk of discrepancies between cohorts which increases the risk of “misclassification”. Since our study cohorts (MEC exempted) included predominantly men of European ancestry, we were limited in our ability to study other ethnicities.

Genome-wide association studies have been particularly successful for prostate cancer. Recently published secondary analysis of GWAS has now added ∼10 additional prostate cancer SNPs to those presented here [5], [9], [11]. At time of this study, we did not have genotype data for these SNPs in BPC3 and it remains to be seen if they are differentially associated with clinical subtypes or if they interact with non-genetic factors.

In summary, we independently replicated the association between prostate cancer risk and 36 SNPs identified in multi-stage genome-wide association studies of prostate cancer. Except for SNPs in *KLK3* that were differentially associated with Gleason grade, we did not detect any differentiation in SNP associations according to Gleason grade or stage at diagnosis, two clinical factors strongly predictive of disease outcome. Moreover, we found no strong evidence that these SNPs interact with age, family history, diabetes, BMI, height, smoking or alcohol consumption.

## Materials and Methods

### Study Population

The BPC3 has been described in detail elsewhere [34]. In brief, the consortium combines resources from seven well-established cohort studies with blood samples collected as follows: the Alpha-Tocopherol, Beta-Carotene Cancer Prevention (ATBC) Study in 1992-1993 [35], American Cancer Society Cancer Prevention Study II (CPS-II) in 1998 [36], the European Prospective Investigation into Cancer and Nutrition Cohort (EPIC – comprised of cohorts from Denmark, Great Britain, Germany, Greece, Italy, the Netherlands, Spain, and Sweden) in 1993 [37], the Health Professionals Follow-up Study (HPFS) in 1993 [38], the Multi-Ethnic Cohort (MEC) in 1995 [39], the Physicians' Health Study (PHS) in 1982 [40], and the Prostate, Lung, Colorectal, and Ovarian (PLCO) Cancer Screening Trial in 1993–2001 [41]. In addition, the Melbourne Collaborative Cohort Study (MCCS) established in 1990–1994 [42] recently joined the consortium. Together, these eight cohorts collectively include over 265,000 men who provided a blood sample.

Prostate cancer cases were identified through population-based cancer registries or self-reports confirmed by medical records, including pathology reports. Except for the MCCS study, the BPC3 consists of a series of matched nested case-control studies within each cohort; controls were matched to cases on a number of potential confounding factors, such as age, ethnicity, and region of recruitment, depending on the cohort. MCCS used a case-cohort design, with a randomly sampled sub-cohort serving as controls. Written informed consent was obtained from all subjects and each study was approved by the Institutional Review Boards at their respective institutions. The IRBs for each study were as follows: US National Cancer Institute and National Institute for Health and Welfare (Helsinki, Finland) (ATBC), The Emory University School of Medicine Institutional Review Board (CPS-II), Ethikkommission - Medizinische Fakultät Heidelberg and Imperial College Research Ethics Committee (EPIC), The Institutional Review Board of Harvard School of Public Health (HPFS), The Cancer Council Victoria Human Research Ethics Committee (MCCS), The Institutional Review Board at the University of Southern California and the Institutional Review Board at the University of Hawaii (MEC), The Human Subjects Committee at Brigham and Women's Hospital (PHS) and NCI Special Studies Institutional Review Board (PLCO).

The current study was restricted to individuals who self-reported as being Caucasian. We had genotype data for a total of 10,501 prostate cancer cases and 10,831 controls. Data on disease stage and grade at time of diagnosis were collected from each cohort, wherever possible. A total of 1,717 cases were classified as high-stage (stage C or D at diagnosis) and 1,388 were classified as high-grade (Gleason grade >7 or equivalent, i.e. coded as poorly differentiated or undifferentiated). For 15% of the cases, we did not have information about tumor stage or Gleason grade.

Baseline information of height and body weight, family history of prostate cancer, cigarette smoking status (never, past, and current), alcohol intake (g/day) and information about a pre-existing diabetes diagnosis were collected by self-report. Family history, which was defined as having at least one first-degree family member diagnosed with prostate cancer, was available for all but two cohort studies (PHS and EPIC). For some countries in EPIC, weight and height was measured.

### Collection and harmonization of non-genetic data

We collected data on family history, diabetes at baseline, smoking, alcohol consumption, height and BMI for each study. Family history of prostate cancer was dichotomized into “yes” (1,780 subjects) or “no” (12,382 subjects). Age was calculated at age of diagnosis/selection as control except for MCCS (at baseline for controls) and MEC (at blood draw for controls) and further dichotomized into younger or equal to 65 years old or older than 65 years. BMI was calculated based on baseline weight (kg) and height (m) categorized into 3 categories: normal weight (BMI<25 kg/m^2^, 7,947 subjects), overweight (BMI 25–30 kg/m^2^, 10,206 subjects) and obese (BMI>30 kg/m^2^, 2,771 subjects). Height was analyzed both as a continuous variable and in tertiles (<173 cm (7,221 subjects), 173–180 cm (7,324 subjects) and >180 cm (6,548 subjects).

Smoking was categorized into 3 categories: never (7,725 subjects), former (9,457 subjects) and current (3,989 subjects). Alcohol was dichotomized into never and moderate drinkers (<30 g/day or two drinks per day; 17,398 subjects) or heavy drinkers (≥30 g/day or 2 drinks per day; 3,257 subjects). Pre-existing diabetes was dichotomized into “yes” (982 subjects) or “no” (19,643 subjects).

We agreed on a common protocol prior to data collection based on data availability in the studies. Each study was responsible for sending the data in a format as described in the protocol to facilitate data harmonization. We agreed on collecting as detailed information as possible without having to exclude any study due to lack of covariate information (that is, we aimed for the least common denominator for the variables of interest). Inconsistencies or clarifications were handled by a dialogue between the data coordinating center and the individual studies. All studies have published analysis on these variables earlier and details on quality checks can be found in study-specific publications. All statistical analyses were conducted centrally.

### SNP selection and genotyping

We selected 39 SNPs based on the literature for prostate cancer GWAS (Table 2). These include (genomic location in parenthesis): rs721048 (2p15), rs1465618 (2p21), rs12621278 (2q31.3), rs2660753 (3q12.1), rs4857841 (3q21.3), rs17021918 (4q22.3), rs12500426 (4q22.3), rs7679673 (4q24), rs9364554 (6q25.3), rs10486567 (7p15.2), rs6465657 (7p21.3), rs1512268 (8p21.2), rs2928679 (8p21.2), rs4961199 (8q21.3), rs1016343 (8q24.21), rs7841060 (8q24.21), rs16901979 (8q24.21), rs620861 (8q24.21), rs6983267 (8q24.21), rs1447295 (8q24.21), rs4242382 (8q24.21), rs7837688 (8q24.21), rs16902094 (8q24.21), rs1571801 (9p33.2), rs10993994 (10q11.23), rs4962416 (10q26.13), rs7127900 (11p15.5), rs12418451 (11q13.2), rs7931342 (11q13.2), rs10896449 (11q13.2), rs11649743 (17q12), rs4430796 (17q12), rs7501939 (17q12), rs1859962 (17q24.3), rs266849 (19p13.33), rs2735839 (19p13.33), rs5759167 (22q13.2), rs5945572 (Xp11.22) and rs5945619 (Xp11.22). For rs12418451, we used genotypes from either rs12418451 or rs10896438 (r^2^ = 0.964 in HapMap CEU population) and for and rs2928679 we used genotypes from either and rs2928679 or rs13264338 (r2 = 0.966 in HapMap CEU population). We did not have genotype data on rs4961199, rs16901979 and rs16902094 for MCCS.

Genotyping was performed using the TaqMan assay (Applied Biosystems, Foster City, CA) in five different genotyping laboratories: Core Genotyping Facility at National Cancer Institute, Harvard School of Public Health, University of South California, DKFZ and UK Cancer Research. Blinded duplicated samples indicated no genotyping error. For each autosomal SNP, we tested HWE in the controls in each study separately. All autosomal SNPs were in HWE (P>0.01).

### Statistical methods

We tested the association between prostate cancer risk and each SNP with a likelihood ratio test based on unconditional logistic regression. We adjusted all analyses for study and age at diagnosis or selection as a control in five year intervals using indicator variables. All odds ratios are calculated per copy of the minor alleles (0,1,2) carried. For each SNP, we used Cochran's Q statistic to test for heterogeneity between studies.

To estimate odds ratios for high-grade or low-grade disease, we performed multinomial regression with an outcome variable coded as 0 (control), 1 (low-grade) or 2 (high-grade). To test for differential SNP associations between low-grade and high-grade disease, we used a likelihood ratio test based on case-only analysis. We repeated this analysis for high-stage/low-stage disease.

We tested for interaction between SNPs and non-genetic factors by conducting a one degree-of-freedom likelihood ratio test of a single interaction term (SNPxE) as implemented in an unconditional logistic regression. When an environmental factor had more than two categories (as is the case for smoking, BMI and height), we used ordinal coding for the interaction term. To explore whether associations with proposed environmental risk factors may have been masked by effect heterogeneity, we performed a joint (2 d.f.) test of the environmental main effect and the interaction effect. This test has been shown to outperform the standard marginal test when the environmental effect is restricted to a genetic stratum [21]. Cohorts with no variability in exposure (such as ATBC and smoking) were excluded from gene-environment interaction analyses. We tested for pair-wise SNP-SNP interactions using a one degree-of-freedom likelihood ratio test of a single interaction term as described for the SNP-environment interaction tests.

We tested for dominance deviation from an additive model by including an additional SNP covariate coded as (0,1,0) for (homozygote common allele, heterozygote, homozygote rare allele) respectively. Based on unconditional regression, we performed a one degree-of-freedom likelihood ratio test where the full model was tested against a model only including the SNP covariate with additive coding (0,1,2) as described above. All reported P values are two-sided and uncorrected for multiple hypothesis testing. Analyses were conducted in R [43] and SAS version 9.1.

## Supporting Information

Figure S1 Study-specific SNP associations with prostate cancer risk. For rs4961199, rs16901979 and rs16902094 we did not have genotype data from MCCS.
(DOC)

Table S1 Heterogeneity in main effects between studies for selected SNPs.
(DOC)

Table S2 Associations between SNPs identified through CGEMS and prostate cancer risk stratified on CGEMS membership.
(DOC)

Table S3 SNP-Environment interactions for family history of prostate cancer and age at diagnosis.
(DOC)

Table S4 SNP-Environment interactions with diabetes and BMI.
(DOC)

Table S5 Association between height and prostate cancer risk stratified by SNP genotypes.
(DOC)

Table S6 Association between smoking and prostate cancer risk stratified by SNP genotypes.
(DOC)

Table S7 Association between alcohol consumption and prostate cancer risk stratified by SNP genotypes.
(DOC)

Table S8 Pair-wise SNP-SNP interactions that reached a p-value < 0.05.
(DOC)
