# Supplementary material for: Characterizing Associations and SNP-Environment Interactions for GWAS-Identified Prostate Cancer Risk Markers—Results from BPC3
Source: PLoS One. 2011 Feb 24;6(2):e17142. doi: 10.1371/journal.pone.0017142 (PMC3044744; doi:10.1371/journal.pone.0017142)

**Supplementary Figure 1.** Study-specific SNP associations with prostate cancer risk. For rs4961199, rs16901979 and rs16902094 we did not have genotype data from MCCS.

rs721048 rs1465618


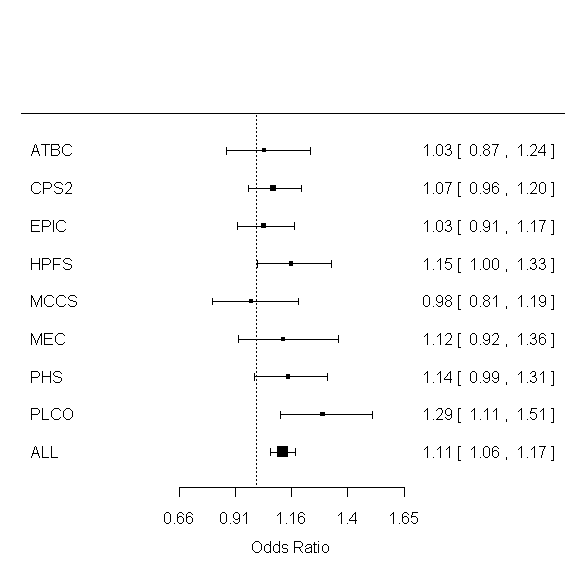

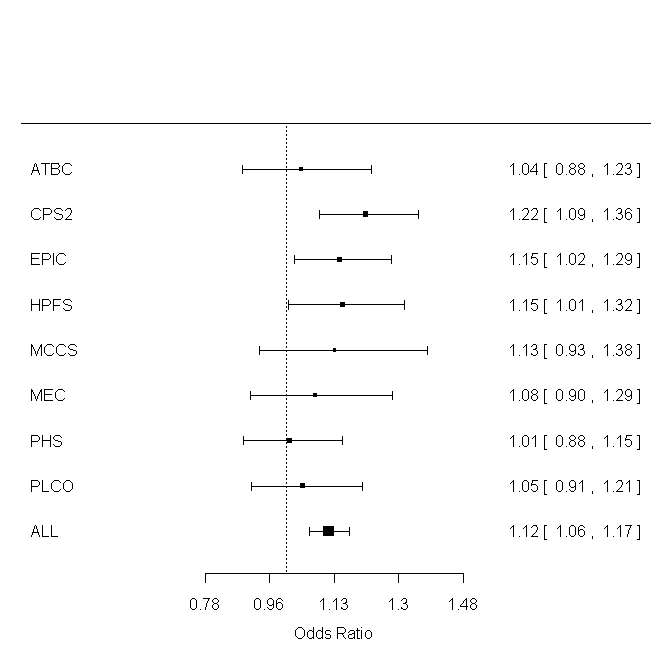


rs12621278 rs2660753


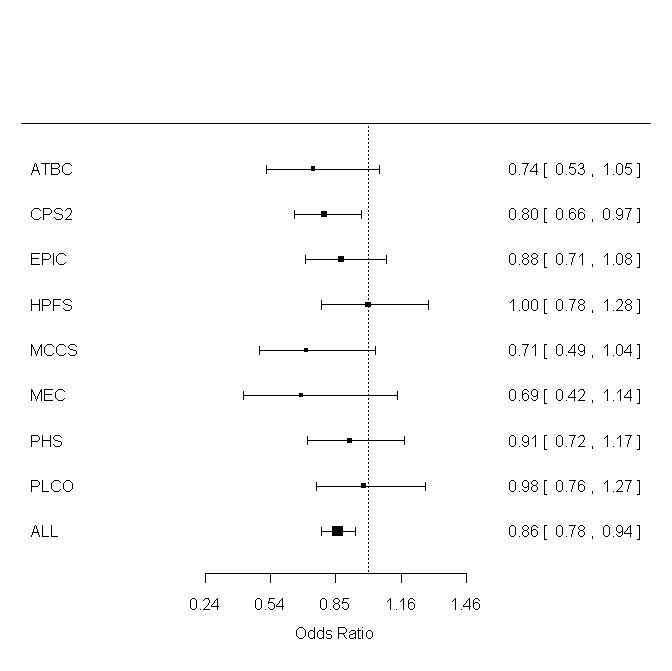

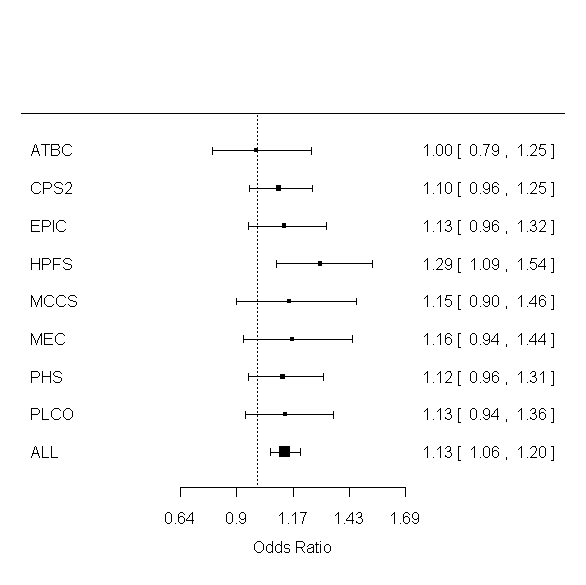


rs4857841 rs17021918


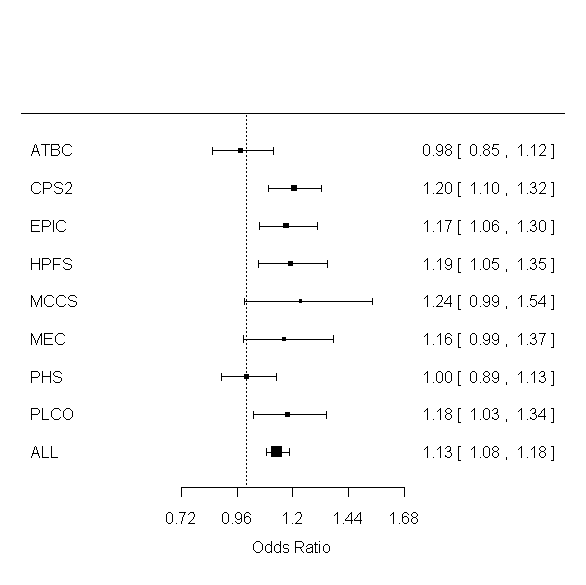

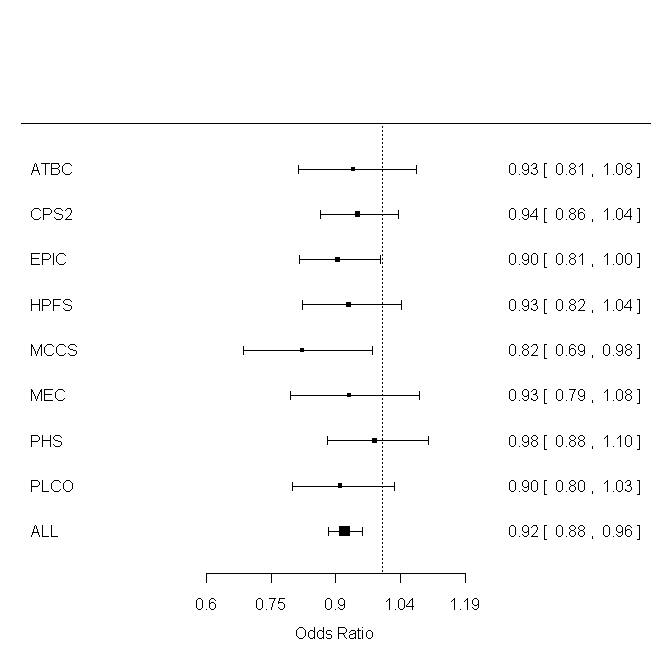


rs12500426 rs7679673


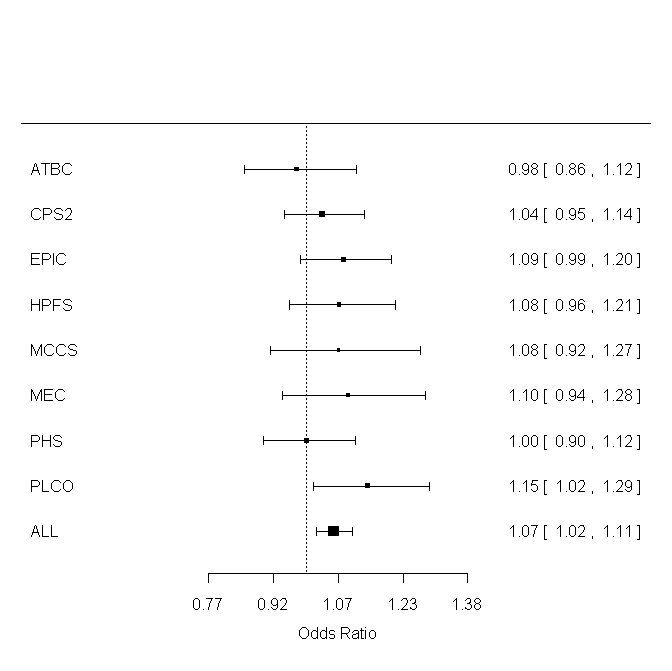

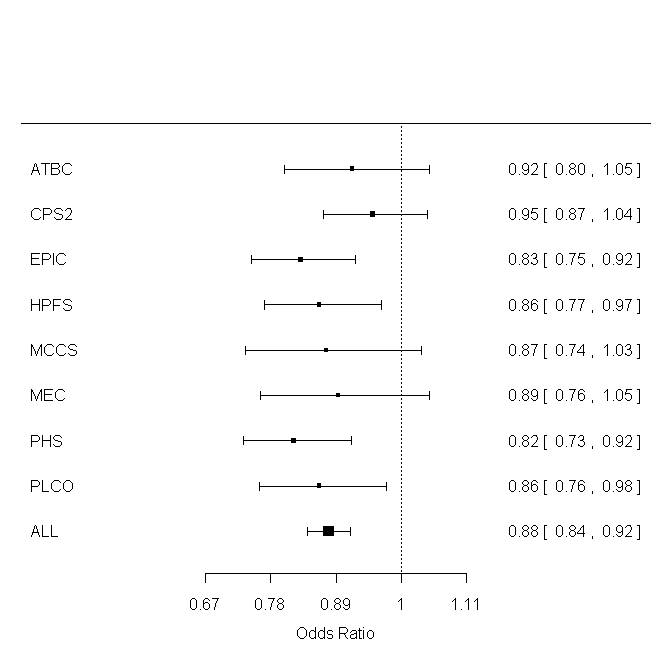


rs9364554 rs10486567


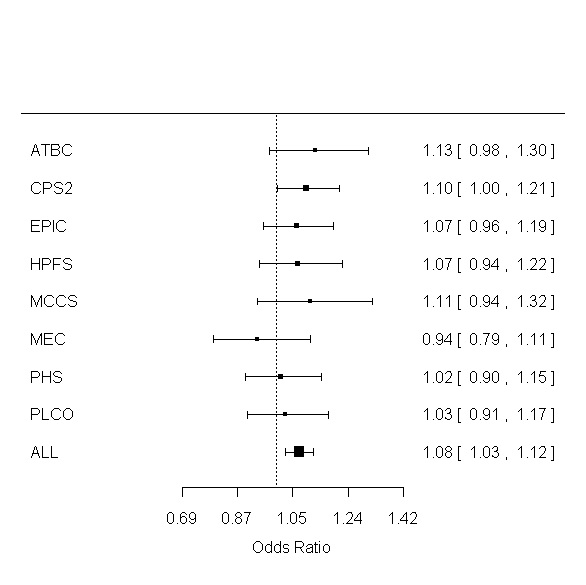

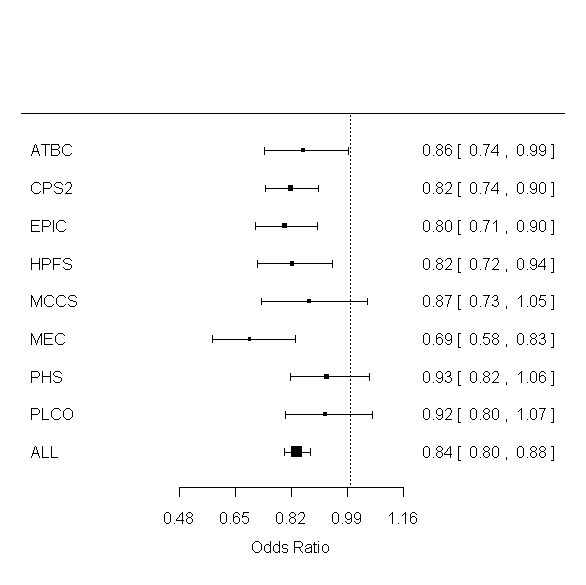


rs6465657 rs1512268


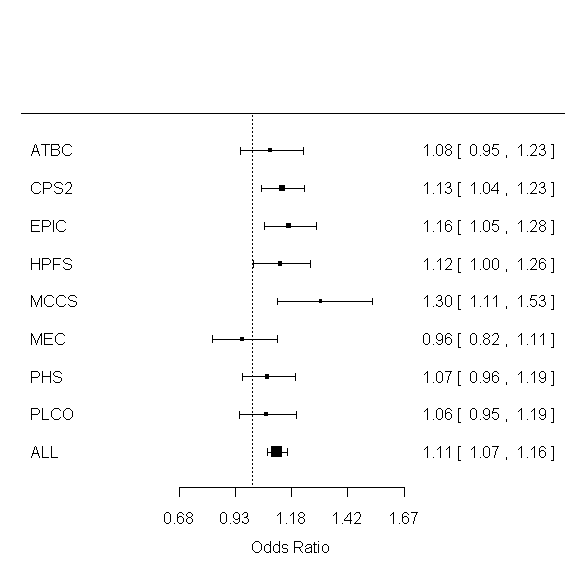

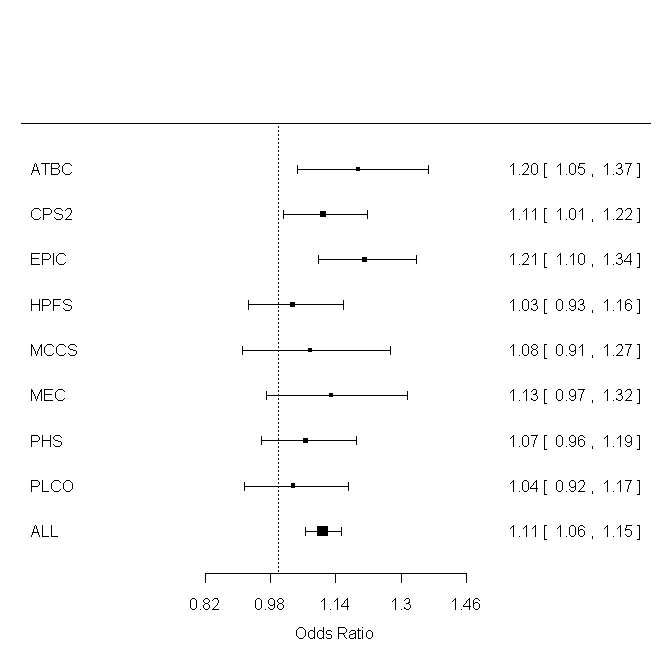


rs2928679 rs4961199


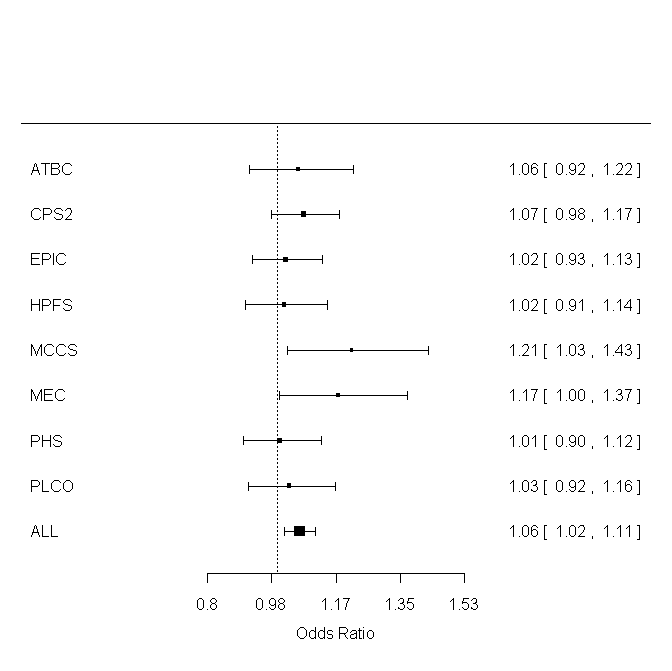

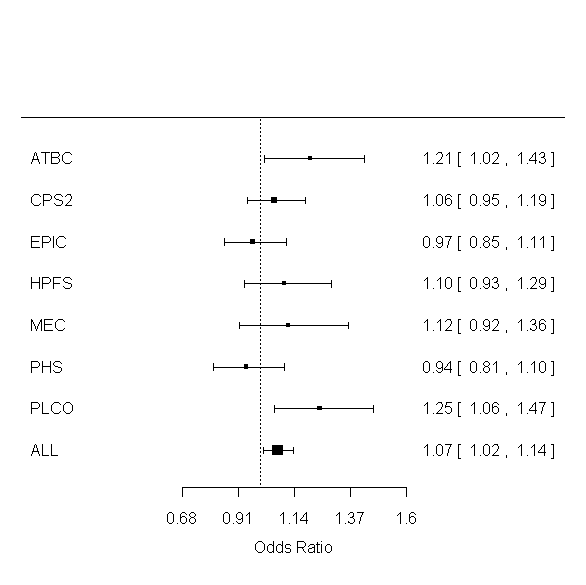


rs1016343 rs7841060


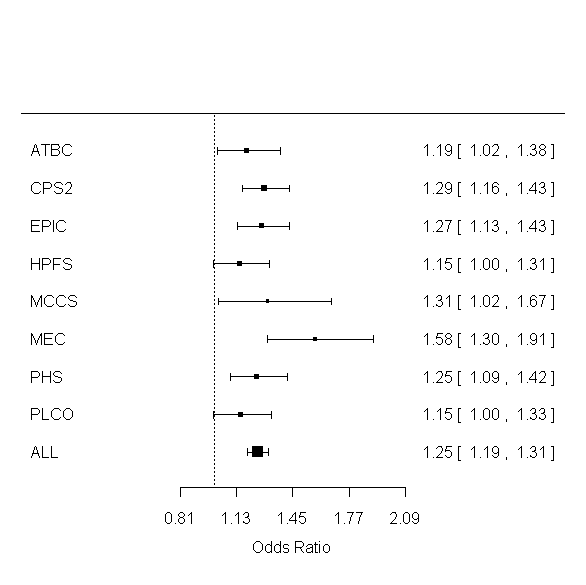

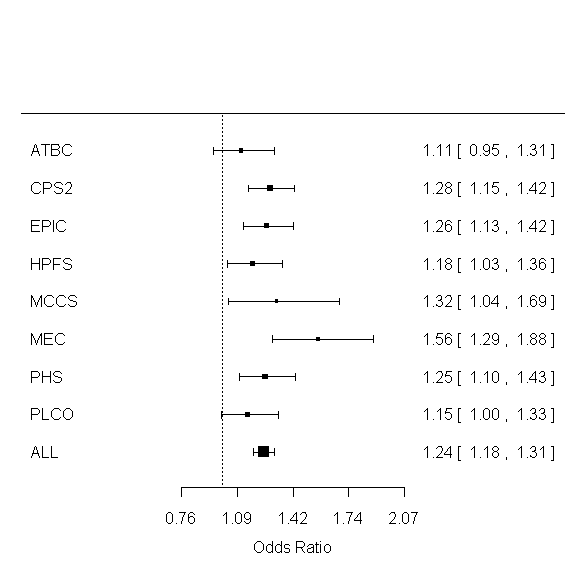


rs16901979 rs620861


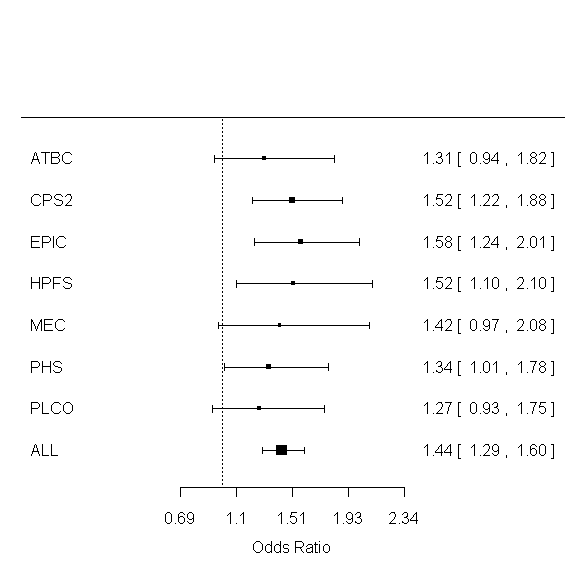

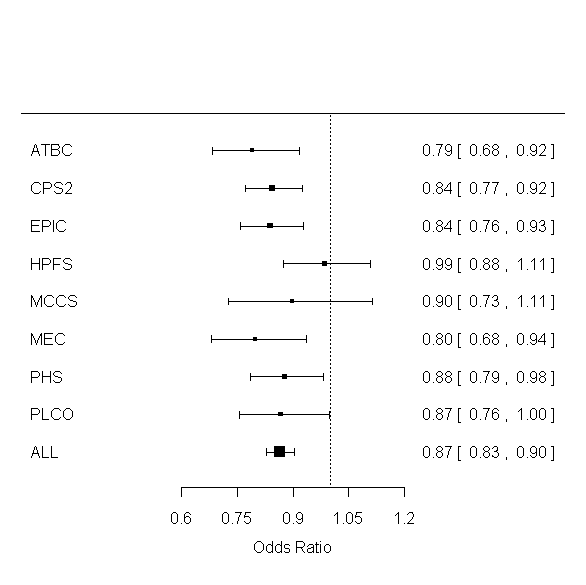


rs6983267 rs1447295


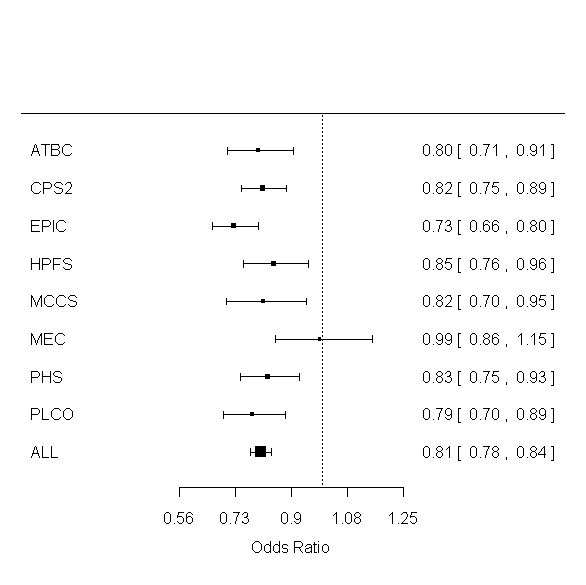

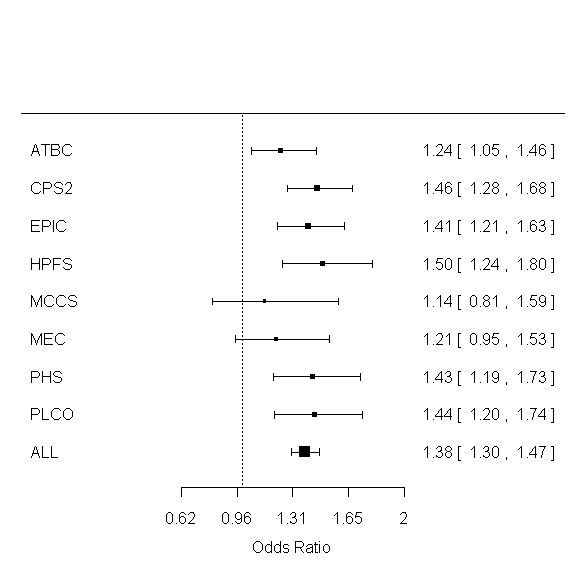


rs4242382 rs7837688


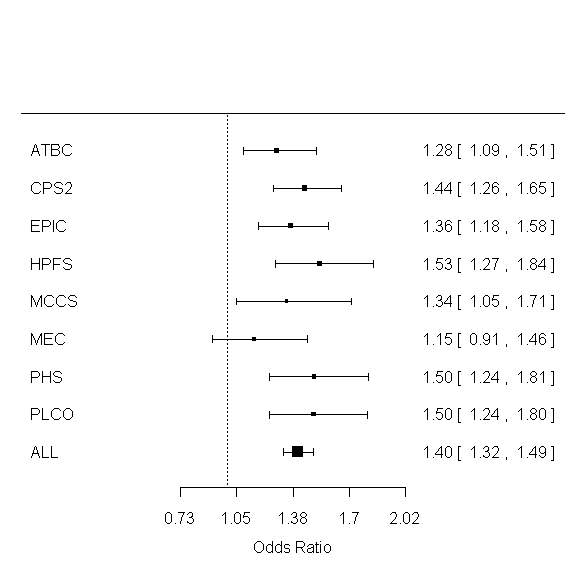

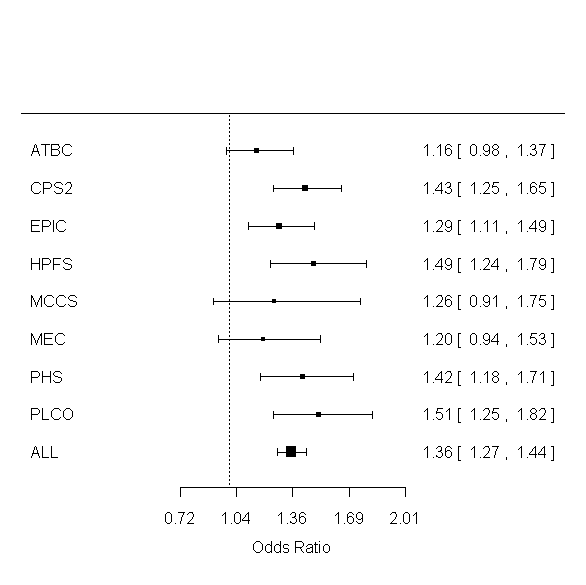


rs16902094 rs1571801


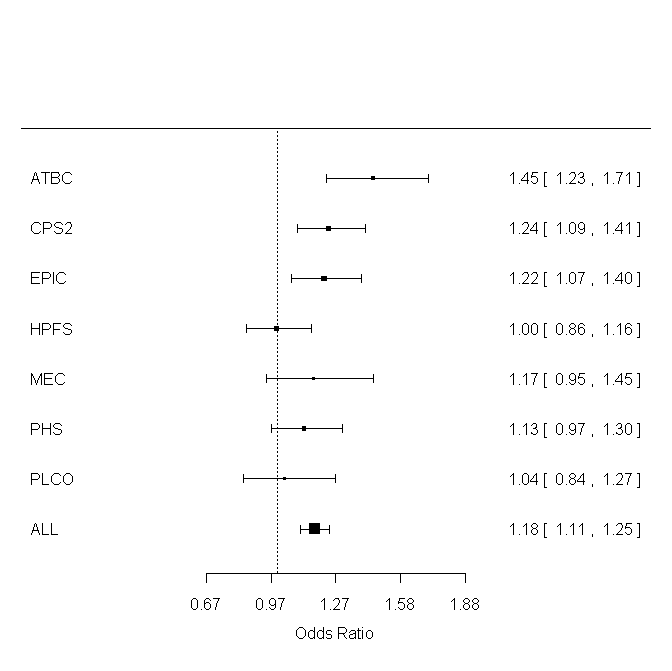

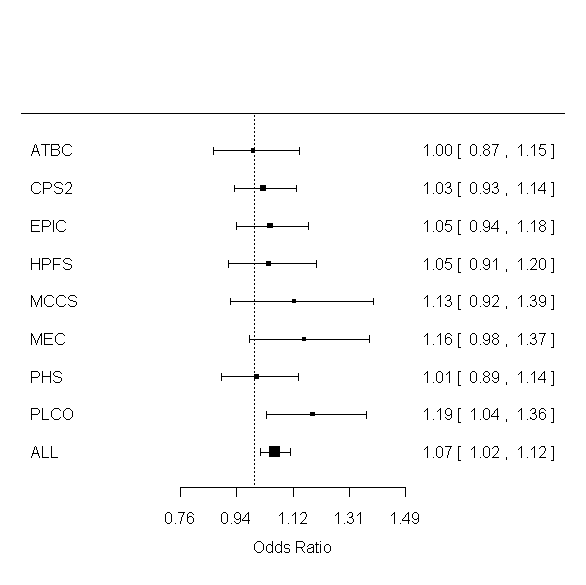


rs10993994 rs4962416


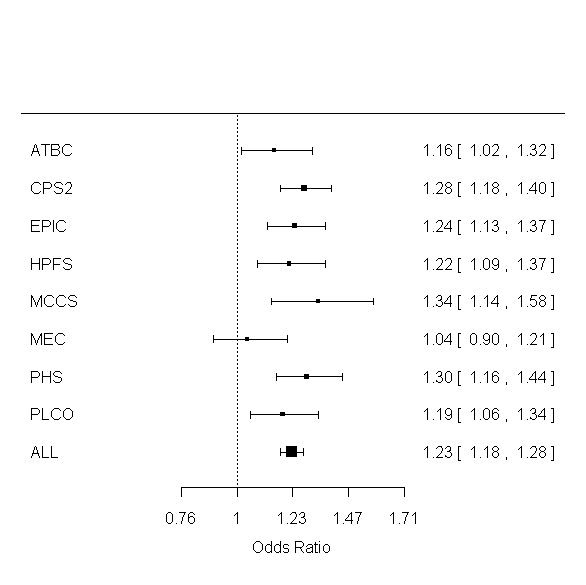

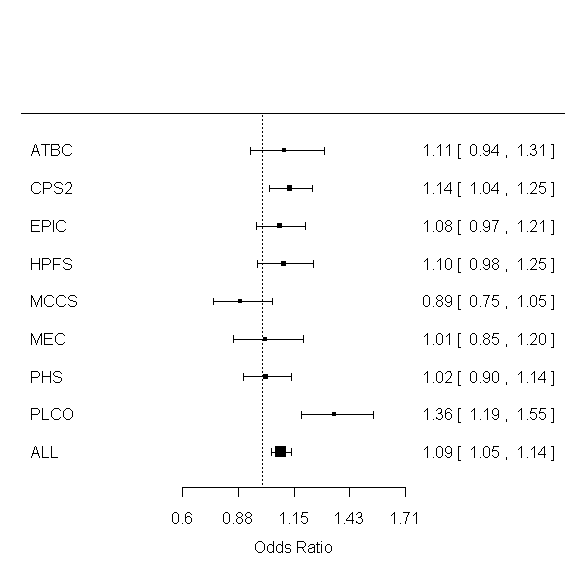


rs7127900 rs12418451


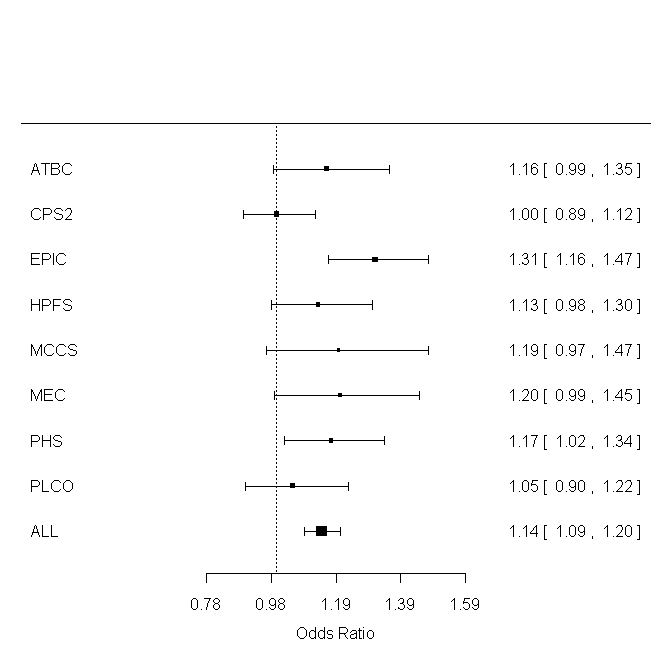

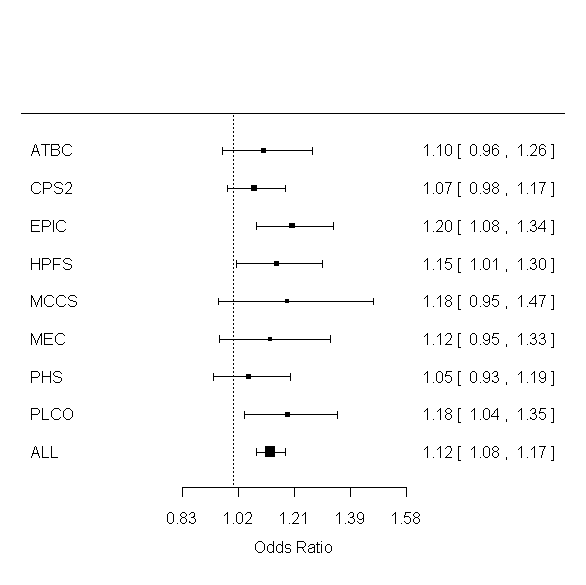


rs7931342 rs10896449


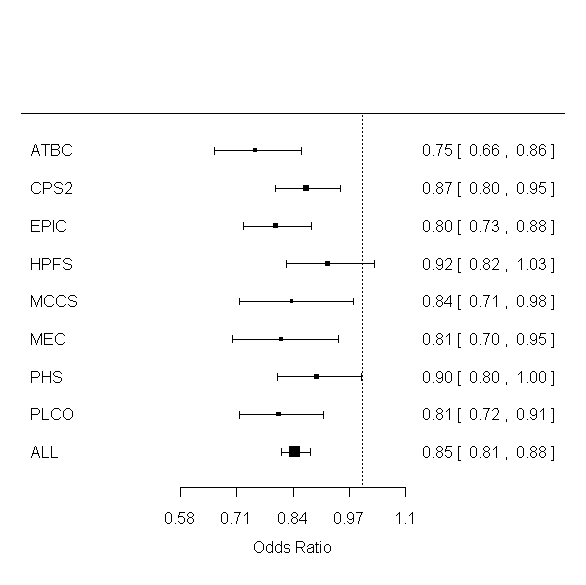

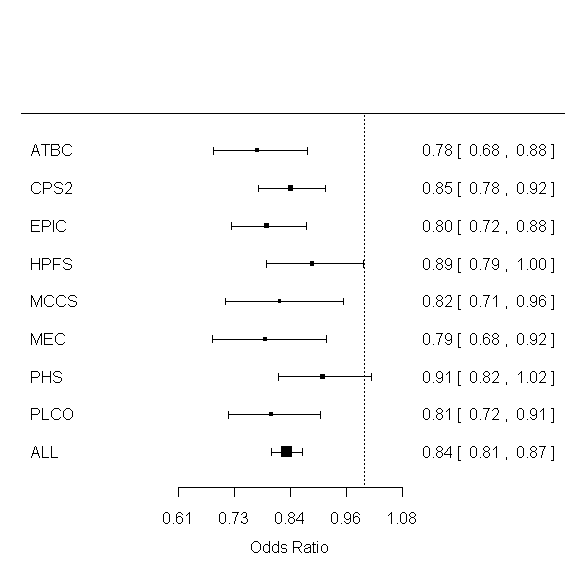


rs11649743 rs4430796


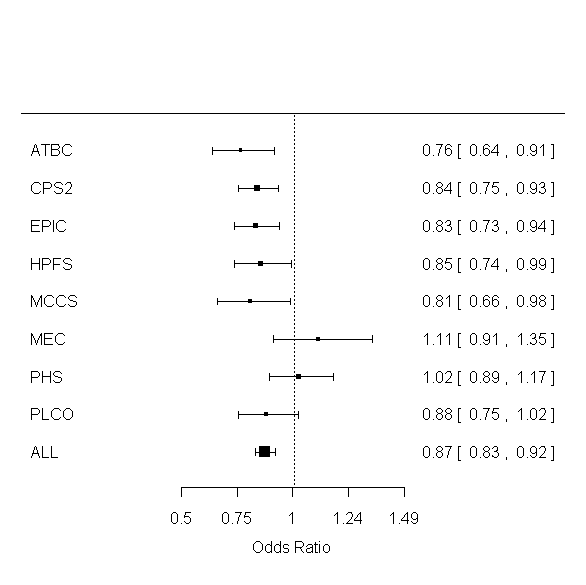

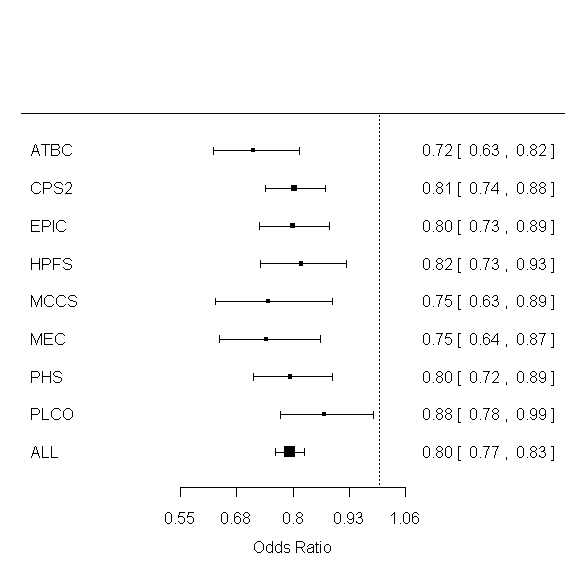


rs7501939 rs1859962


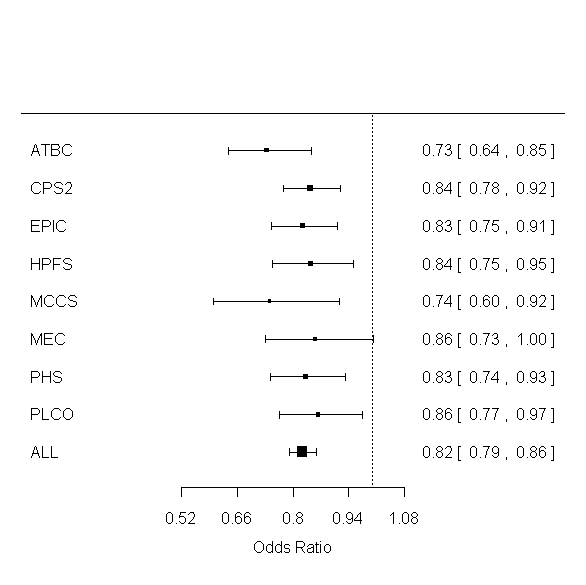

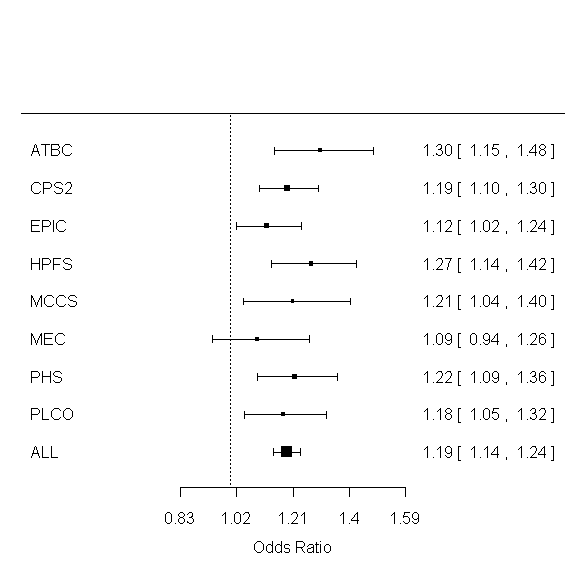


rs266849 rs2735839


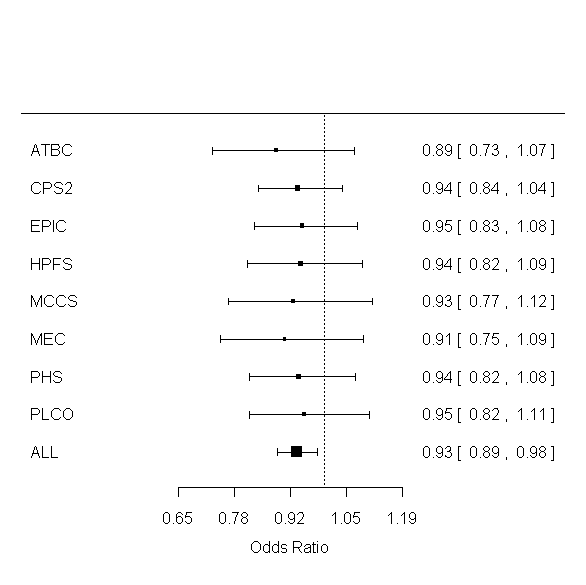

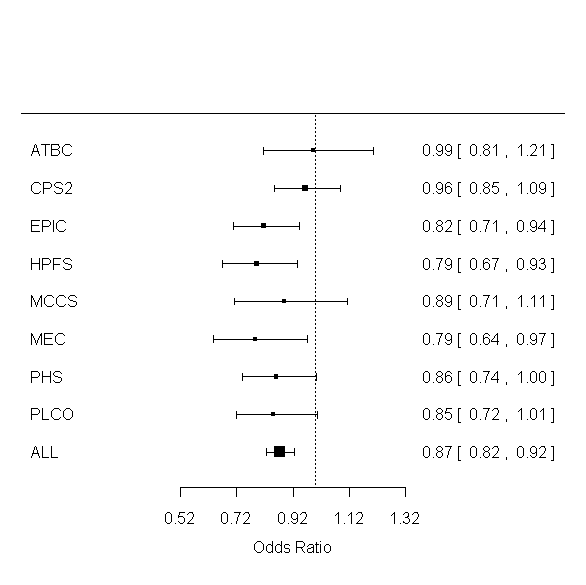


rs5759167 rs5945572


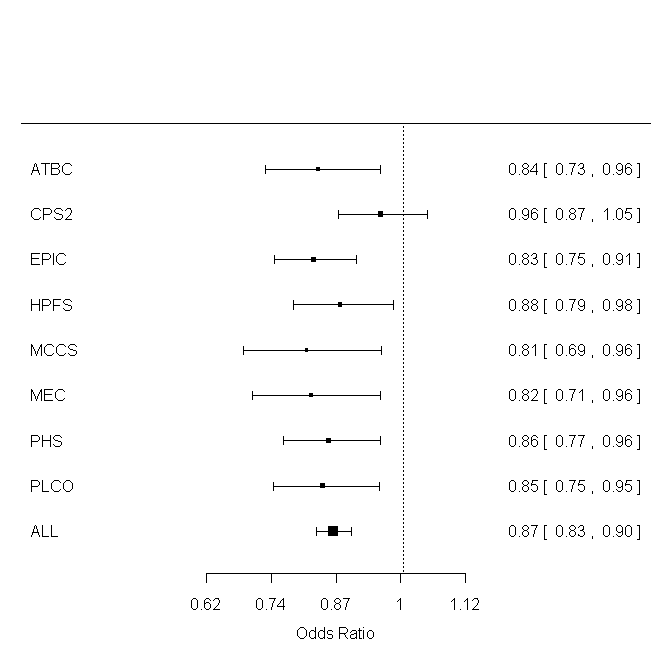

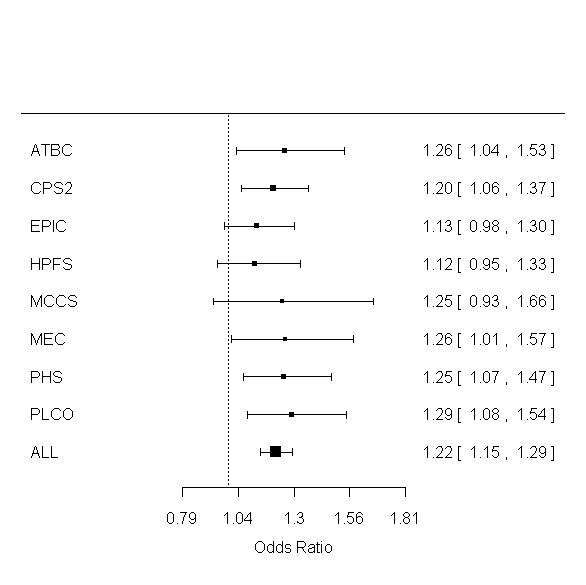


rs5945619


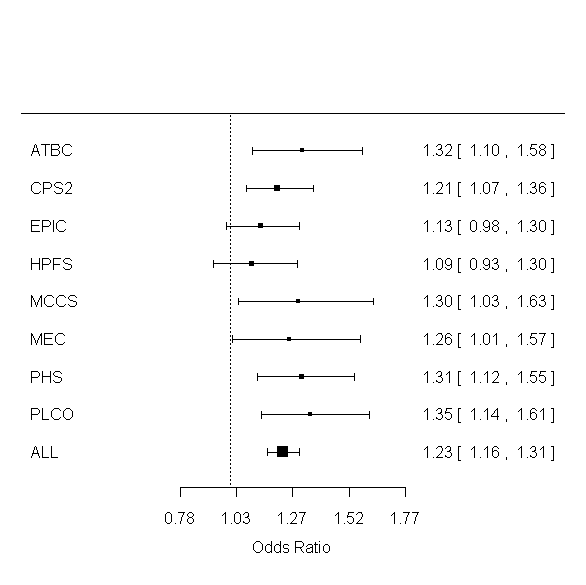

Supplement: Figure S1 — Study-specific SNP associations with prostate cancer risk. For rs4961199, rs16901979 and rs16902094 we did not have genotype data from MCCS. (DOC) [file pone.0017142.s001.doc]
