# Supplementary material for: Characterizing Associations and SNP-Environment Interactions for GWAS-Identified Prostate Cancer Risk Markers—Results from BPC3
Source: PLoS One. 2011 Feb 24;6(2):e17142. doi: 10.1371/journal.pone.0017142 (PMC3044744; doi:10.1371/journal.pone.0017142)
Supplement: Table S1 — Heterogeneity in main effects between studies for selected SNPs. (DOC) [file pone.0017142.s002.doc]

**Supplementary Table 1:** Heterogeneity in main effects between studies for selected SNPs.

| SNP | Q | P | I^2^ (%) |
| --- | --- | --- | --- |
| rs721048 | 7.95 | 0.34 | 11.90 |
| rs1465618 | 6.62 | 0.47 | 0 |
| rs12621278 | 5.73 | 0.57 | 0 |
| rs2660753 | 3.75 | 0.81 | 0 |
| rs4857841 | 12.60 | 0.08 | 44.44 |
| rs17021918 | 3.53 | 0.83 | 0 |
| rs12500426 | 4.85 | 0.68 | 0 |
| rs7679673 | 6.18 | 0.52 | 0 |
| rs9364554 | 4.33 | 0.74 | 0 |
| rs10486567 | 9.61 | 0.21 | 27.16 |
| rs6465657 | 10.01 | 0.19 | 30.09 |
| rs1512268 | 7.64 | 0.37 | 8.37 |
| rs2928679 | 6.18 | 0.52 | 0 |
| rs4961199 | 10.24 | 0.12 | 41.39 |
| rs1016343 | 9.60 | 0.21 | 27.10 |
| rs7841060 | 9.71 | 0.21 | 27.89 |
| rs16901979 | 2.01 | 0.92 | 0 |
| rs620861 | 7.79 | 0.35 | 10.18 |
| rs6983267 | 12.86 | 0.08 | 45.57 |
| rs1447295 | 6.08 | 0.53 | 0 |
| rs4242382 | 6.04 | 0.53 | 0 |
| rs7837688 | 8.35 | 0.30 | 16.16 |
| rs16902094 | 13.53 | 0.04 | 55.66 |
| rs1571801 | 6.05 | 0.53 | 0 |
| rs10993994 | 8.96 | 0.26 | 21.87 |
| rs4962416 | 19.67 | 0.01 | 64.41 |
| rs7127900 | 12.06 | 0.10 | 41.97 |
| rs12418451 | 4.44 | 0.73 | 0 |
| rs7931342 | 8.73 | 0.27 | 19.79 |
| rs10896449 | 7.09 | 0.42 | 1.28 |
| rs11649743 | 14.73 | 0.04 | 52.48 |
| rs4430796 | 6.41 | 0.49 | 0 |
| rs7501939 | 4.73 | 0.69 | 0 |
| rs1859962 | 6.22 | 0.51 | 0 |
| rs266849 | 0.55 | 1.00 | 0 |
| rs2735839 | 7.39 | 0.39 | 5.34 |
| rs5759167 | 7.15 | 0.41 | 2.14 |
| rs5945572 | 2.73 | 0.91 | 0 |
| rs5945619 | 5.79 | 0.56 | 0 |
