# Supplementary material for: Characterizing Associations and SNP-Environment Interactions for GWAS-Identified Prostate Cancer Risk Markers—Results from BPC3
Source: PLoS One. 2011 Feb 24;6(2):e17142. doi: 10.1371/journal.pone.0017142 (PMC3044744; doi:10.1371/journal.pone.0017142)
Supplement: Table S2 — Associations between SNPs identified through CGEMS and prostate cancer risk stratified on CGEMS membership. (DOC) [file pone.0017142.s003.doc]

**Supplementary Table 2:** Associations between SNPs identified through CGEMS and prostate cancer risk stratified on CGEMS membership.

|  |  |  | CGEMS | | NON-CGEMS^1^ | | NON-CGEMS^2^ | |
| --- | --- | --- | --- | --- | --- | --- | --- | --- |
| SNP | Chr | Gene/Region | OR (95% CI) | P | OR (95% CI) | P | OR (95% CI) | P |
| rs4857841 | 3 | *EEFSEC* | 1.16 (1.10-1.21) | 6.6∙10^-9^ |  |  | 1.03 (0.93-1.15) | 0.52 |
| rs10486567 | 7 | *JAZF1* | 0.85 (0.79-0.90) | 1.6∙10^-7^ | 0.83 (0.77-0.89) | 8.7∙10^-8^ |  |  |
| rs4961199 | 8 | *CPNE3,CNGB3* | 1.13 (1.05-1.22) | 0.00064 | 0.99 (0.91-1.08) | 0.82 |  |  |
| rs7841060 | 8 | *--* | 1.24 (1.17-1.31) | 9.9∙10^-15^ |  |  | 1.25 (1.12-1.40) | 8.8∙10^-5^ |
| rs620861 | 8 | *--* | 0.86 (0.82-0.90) | 5.4∙10^-10^ |  |  | 0.89 (0.80-0.98) | 0.014 |
| rs6983267 | 8 | *--* | 0.81 (0.77-0.86) | 1.9∙10^-14^ | 0.81 (0.77-0.86) | 1.6∙10^-6^ |  |  |
| rs4242382 | 8 | *--* | 1.43 (1.32-1.55) | 2.2∙10^-18^ | 1.36 (1.24-1.50) | 4.2∙10^-11^ |  |  |
| rs7837688 | 8 | *--* | 1.39 (1.28-1.50) | 2.8∙10^-15^ | 1.31 (1.19-1.45) | 3.9∙10^-8^ |  |  |
| rs10993994 | 10 | *MSMB* | 1.23 (1.17-1.30) | 2.0∙10^-14^ | 1.23 (1.16-1.30) | 8.1∙10^-12^ |  |  |
| rs4962416 | 10 | *CTBP2* | 1.17 (1.10-1.24) | 4.1∙10^-7^ | 1.01 (0.95-1.08) | 0.73 |  |  |
| rs10896449 | 11 | *--* | 0.84 (0.79-0.88) | 3.4∙10^-11^ | 0.84 (0.79-0.89) | 1.6∙10^-9^ |  |  |

^1^ Studies not included in CGEMS Stage 2: EPIC, MCCS, MEC, PHS: 4,661 cases and 5,288 controls

^2^ Studies not included in CGEMS Stage 3: MCCS, PHS: 2,700 cases and 2,412 controls
