# Supplementary material for: Characterizing Associations and SNP-Environment Interactions for GWAS-Identified Prostate Cancer Risk Markers—Results from BPC3
Source: PLoS One. 2011 Feb 24;6(2):e17142. doi: 10.1371/journal.pone.0017142 (PMC3044744; doi:10.1371/journal.pone.0017142)
Supplement: Table S3 — SNP-Environment interactions for family history of prostate cancer and age at diagnosis. (DOC) [file pone.0017142.s004.doc]

**Supplementary Table 3:** SNP-Environment interactions for family history of prostate cancer and age at diagnosis.

|  | OR (95% CI) | |  | OR (95% CI) | |  |
| --- | --- | --- | --- | --- | --- | --- |
| SNP | FH + | FH - | P^1^ | ≤ 65 years | > 65 years | P^1^ |
| rs721048 | 1.11 (0.92-1.33) | 1.13 (1.06-1.21) | 0.98 | 1.09 (1.00-1.19) | 1.11 (1.04-1.18) | 0.85 |
| rs1465618 | 1.14 (0.96-1.36) | 1.12 (1.05-1.19) | 0.82 | 1.10 (1.01-1.19) | 1.12 (1.06-1.19) | 0.62 |
| rs12621278 | 0.75 (0.53-1.05) | 0.89 (0.79-1.00) | 0.30 | 0.84 (0.72-0.98) | 0.87 (0.78-0.98) | 0.70 |
| rs2660753 | 1.11 (0.89-1.38) | 1.14 (1.05-1.23) | 0.71 | 1.18 (1.06-1.31) | 1.10 (1.02-1.19) | 0.29 |
| rs17021918 | 0.92 (0.78-1.07) | 0.92 (0.87-0.97) | 0.86 | 0.85 (0.79-0.91) | 0.96 (0.91-1.01) | 0.01 |
| rs12500426 | 1.05 (0.91-1.21) | 1.07 (1.01-1.13) | 0.85 | 1.10 (1.03-1.18) | 1.05 (1.00-1.10) | 0.17 |
| rs7679673 | 0.87 (0.75-1.01) | 0.89 (0.84-0.94) | 0.59 | 0.87 (0.81-0.93) | 0.88 (0.84-0.93) | 0.96 |
| rs9364554 | 1.12 (0.96-1.31) | 1.08 (1.02-1.14) | 0.42 | 1.15 (1.07-1.24) | 1.03 (0.98-1.09) | 0.06 |
| rs10486567 | 0.74 (0.63-0.87) | 0.83 (0.78-0.88) | 0.15 | 0.86 (0.79-0.93) | 0.82 (0.78-0.87) | 0.36 |
| rs6465657 | 1.08 (0.94-1.24) | 1.11 (1.05-1.17) | 0.71 | 1.16 (1.09-1.25) | 1.08 (1.03-1.14) | 0.13 |
| rs1512268 | 1.11 (0.96-1.29) | 1.08 (1.02-1.14) | 0.68 | 1.18 (1.10-1.26) | 1.07 (1.02-1.13) | 0.03 |
| rs2928679 | 1.13 (0.97-1.31) | 1.07 (1.01-1.13) | 0.50 | 1.06 (0.99-1.14) | 1.05 (1.00-1.11) | 0.55 |
| rs1016343 | 1.14 (0.96-1.35) | 1.26 (1.18-1.34) | 0.27 | 1.27 (1.17-1.38) | 1.24 (1.17-1.32) | 0.76 |
| rs7841060 | 1.10 (0.92-1.30) | 1.26 (1.18-1.34) | 0.12 | 1.26 (1.16-1.37) | 1.24 (1.17-1.32) | 0.88 |
| rs16901979 | 1.65 (1.11-2.47) | 1.39 (1.20-1.60) | 0.43 | 1.63 (1.34-1.99) | 1.38 (1.21-1.56) | 0.16 |
| rs620861 | 0.88 (0.75-1.03) | 0.87 (0.82-0.92) | 0.75 | 0.81 (0.75-0.87) | 0.89 (0.85-0.94) | 0.05 |
| rs6983267 | 0.85 (0.74-0.98) | 0.83 (0.79-0.87) | 0.85 | 0.75 (0.70-0.80) | 0.84 (0.80-0.89) | 0.004 |
| rs1447295 | 1.31 (1.05-1.64) | 1.39 (1.28-1.51) | 0.81 | 1.42 (1.28-1.58) | 1.36 (1.26-1.47) | 0.36 |
| rs4242382 | 1.38 (1.10-1.72) | 1.42 (1.31-1.54) | 0.94 | 1.43 (1.29-1.58) | 1.37 (1.27-1.48) | 0.42 |
| rs7837688 | 1.39 (1.11-1.75) | 1.38 (1.27-1.50) | 0.78 | 1.38 (1.24-1.54) | 1.34 (1.24-1.44) | 0.55 |
| rs16902094 | 1.16 (0.94-1.44) | 1.18 (1.09-1.27) | 0.65 | 1.28 (1.16-1.43) | 1.13 (1.05-1.21) | 0.10 |
| rs1571801 | 1.26 (1.06-1.50) | 1.06 (1.00-1.12) | 0.04 | 1.08 (1.00-1.17) | 1.05 (0.99-1.11) | 0.45 |
| rs10993994 | 1.28 (1.11-1.48) | 1.21 (1.15-1.27) | 0.44 | 1.24 (1.16-1.33) | 1.23 (1.17-1.29) | 0.67 |
| rs7127900 | 1.05 (0.88-1.26) | 1.10 (1.03-1.18) | 0.57 | 1.19 (1.10-1.30) | 1.11 (1.04-1.18) | 0.10 |
| rs12418451 | 1.09 (0.94-1.28) | 1.13 (1.07-1.20) | 0.78 | 1.11 (1.03-1.20) | 1.13 (1.07-1.19) | 0.77 |
| rs7931342 | 0.88 (0.77-1.01) | 0.84 (0.80-0.89) | 0.71 | 0.84 (0.78-0.90) | 0.85 (0.81-0.89) | 0.79 |
| rs10896449 | 0.88 (0.77-1.01) | 0.83 (0.79-0.87) | 0.57 | 0.83 (0.77-0.89) | 0.84 (0.80-0.88) | 0.65 |
| rs11649743 | 0.76 (0.64-0.92) | 0.87 (0.81-0.93) | 0.19 | 0.83 (0.76-0.90) | 0.89 (0.84-0.95) | 0.31 |
| rs4430796 | 0.84 (0.72-0.97) | 0.80 (0.76-0.84) | 0.5 | 0.79 (0.74-0.85) | 0.80 (0.76-0.84) | 0.77 |
| rs7501939 | 0.85 (0.73-0.98) | 0.82 (0.78-0.87) | 0.77 | 0.81 (0.76-0.88) | 0.83 (0.79-0.87) | 0.86 |
| rs1859962 | 1.18 (1.03-1.35) | 1.21 (1.15-1.27) | 0.9 | 1.22 (1.14-1.31) | 1.18 (1.13-1.24) | 0.25 |
| rs266849 | 1.02 (0.86-1.23) | 0.92 (0.86-0.98) | 0.21 | 0.92 (0.84-1.00) | 0.95 (0.89-1.01) | 0.29 |
| rs2735839 | 0.82 (0.66-1.01) | 0.91 (0.84-0.98) | 0.5 | 0.82 (0.74-0.91) | 0.90 (0.84-0.96) | 0.15 |
| rs5759167 | 0.89 (0.77-1.03) | 0.87 (0.83-0.92) | 0.81 | 0.84 (0.78-0.90) | 0.88 (0.84-0.92) | 0.39 |
| rs5945572 | 1.15 (0.93-1.43) | 1.26 (1.17-1.37) | 0.44 | 1.26 (1.14-1.40) | 1.19 (1.11-1.28) | 0.45 |
| rs5945619 | 1.15 (0.94-1.41) | 1.26 (1.17-1.36) | 0.42 | 1.28 (1.16-1.42) | 1.20 (1.12-1.29) | 0.42 |

^1^ The p-values correspond to a one-degree of freedom likelihood ratio test of the interaction term as implemented in a logistic regression. FH -: no family history of prostate cancer; FH+: family history of prostate cancer
