# Supplementary material for: Characterizing Associations and SNP-Environment Interactions for GWAS-Identified Prostate Cancer Risk Markers—Results from BPC3
Source: PLoS One. 2011 Feb 24;6(2):e17142. doi: 10.1371/journal.pone.0017142 (PMC3044744; doi:10.1371/journal.pone.0017142)
Supplement: Table S4 — SNP-Environment interactions with diabetes and BMI. (DOC) [file pone.0017142.s005.doc]

**Supplementary Table 4:** SNP-Environment interactions with diabetes and BMI.

|  | OR (95% CI) | |  | OR (95% CI) | | |  |
| --- | --- | --- | --- | --- | --- | --- | --- |
| SNP | Diabetic | Non-Diabetic | P^1^ | BMI < 25 | BMI 25-30 | BMI >30 | P^1^ |
| rs721048 | 1.42 (1.11-1.83) | 1.10 (1.04-1.16) | 0.06 | 1.13 (1.04-1.23) | 1.11 (1.03-1.19) | 1.11 (0.97-1.28) | 0.82 |
| rs1465618 | 0.98 (0.78-1.23) | 1.12 (1.06-1.18) | 0.32 | 1.09 (1.01-1.19) | 1.11 (1.04-1.19) | 1.20 (1.05-1.37) | 0.36 |
| rs12621278 | 0.68 (0.43-1.07) | 0.87 (0.79-0.96) | 0.23 | 0.83 (0.72-0.96) | 0.83 (0.73-0.95) | 1.03 (0.79-1.34) | 0.36 |
| rs2660753 | 1.33 (1.00-1.77) | 1.12 (1.05-1.19) | 0.31 | 1.15 (1.04-1.27) | 1.11 (1.02-1.21) | 1.10 (0.92-1.32) | 0.62 |
| rs17021918 | 0.99 (0.81-1.20) | 0.91 (0.87-0.95) | 0.40 | 0.95 (0.89-1.02) | 0.89 (0.84-0.95) | 0.93 (0.83-1.05) | 0.39 |
| rs12500426 | 1.04 (0.86-1.26) | 1.07 (1.02-1.11) | 0.82 | 1.01 (0.95-1.08) | 1.11 (1.04-1.17) | 1.07 (0.96-1.20) | 0.09 |
| rs7679673 | 0.82 (0.67-1.00) | 0.88 (0.85-0.92) | 0.53 | 0.88 (0.82-0.94) | 0.88 (0.83-0.94) | 0.90 (0.80-1.01) | 0.70 |
| rs9364554 | 1.02 (0.83-1.26) | 1.08 (1.03-1.13) | 0.76 | 1.09 (1.02-1.17) | 1.06 (1.00-1.13) | 1.07 (0.95-1.21) | 0.69 |
| rs10486567 | 0.66 (0.52-0.82) | 0.85 (0.81-0.89) | 0.04 | 0.89 (0.82-0.96) | 0.84 (0.78-0.90) | 0.76 (0.66-0.86) | 0.03 |
| rs6465657 | 1.21 (1.01-1.46) | 1.10 (1.06-1.15) | 0.29 | 1.08 (1.02-1.16) | 1.13 (1.06-1.19) | 1.15 (1.03-1.29) | 0.32 |
| rs1512268 | 0.97 (0.80-1.18) | 1.11 (1.06-1.16) | 0.25 | 1.14 (1.07-1.22) | 1.09 (1.03-1.15) | 1.11 (0.99-1.24) | 0.44 |
| rs2928679 | 1.10 (0.91-1.33) | 1.05 (1.01-1.10) | 0.44 | 1.02 (0.96-1.09) | 1.09 (1.03-1.16) | 1.07 (0.96-1.20) | 0.28 |
| rs1016343 | 1.26 (1.00-1.58) | 1.23 (1.17-1.30) | 0.92 | 1.26 (1.17-1.37) | 1.26 (1.18-1.36) | 1.15 (1.01-1.32) | 0.31 |
| rs7841060 | 1.22 (0.97-1.54) | 1.23 (1.17-1.30) | 0.94 | 1.27 (1.18-1.38) | 1.25 (1.17-1.35) | 1.12 (0.98-1.28) | 0.16 |
| rs16901979 | 1.69 (1.04-2.76) | 1.41 (1.26-1.58) | 0.60 | 1.42 (1.19-1.68) | 1.45 (1.24-1.70) | 1.41 (1.04-1.90) | 0.99 |
| rs620861 | 0.82 (0.66-1.01) | 0.87 (0.83-0.91) | 0.51 | 0.87 (0.81-0.94) | 0.87 (0.82-0.93) | 0.80 (0.71-0.91) | 0.37 |
| rs6983267 | 0.81 (0.68-0.98) | 0.81 (0.78-0.85) | 0.98 | 0.86 (0.80-0.91) | 0.81 (0.76-0.86) | 0.73 (0.66-0.82) | 0.02 |
| rs1447295 | 1.57 (1.17-2.10) | 1.36 (1.28-1.46) | 0.40 | 1.31 (1.18-1.45) | 1.46 (1.33-1.60) | 1.31 (1.11-1.56) | 0.54 |
| rs4242382 | 1.69 (1.26-2.26) | 1.38 (1.30-1.47) | 0.21 | 1.33 (1.20-1.47) | 1.46 (1.34-1.60) | 1.35 (1.15-1.60) | 0.55 |
| rs7837688 | 1.45 (1.08-1.95) | 1.34 (1.26-1.43) | 0.62 | 1.29 (1.17-1.43) | 1.42 (1.30-1.55) | 1.31 (1.10-1.55) | 0.54 |
| rs16902094 | 1.23 (0.93-1.63) | 1.18 (1.11-1.25) | 0.62 | 1.10 (1.00-1.21) | 1.20 (1.10-1.31) | 1.40 (1.18-1.66) | 0.01 |
| rs1571801 | 1.18 (0.95-1.46) | 1.07 (1.02-1.12) | 0.50 | 1.04 (0.96-1.12) | 1.08 (1.01-1.15) | 1.10 (0.97-1.25) | 0.36 |
| rs10993994 | 1.31 (1.09-1.58) | 1.23 (1.18-1.28) | 0.66 | 1.25 (1.17-1.33) | 1.23 (1.16-1.30) | 1.22 (1.09-1.36) | 0.57 |
| rs7127900 | 1.18 (0.94-1.48) | 1.14 (1.09-1.20) | 0.67 | 1.13 (1.04-1.22) | 1.16 (1.08-1.25) | 1.13 (0.98-1.30) | 0.80 |
| rs12418451 | 1.31 (1.06-1.61) | 1.11 (1.06-1.16) | 0.19 | 1.09 (1.01-1.17) | 1.16 (1.08-1.23) | 1.13 (1.00-1.28) | 0.32 |
| rs7931342 | 0.81 (0.67-0.97) | 0.85 (0.82-0.89) | 0.81 | 0.87 (0.81-0.93) | 0.81 (0.77-0.86) | 0.88 (0.79-0.98) | 0.73 |
| rs10896449 | 0.79 (0.66-0.95) | 0.84 (0.81-0.88) | 0.66 | 0.86 (0.81-0.92) | 0.81 (0.76-0.86) | 0.87 (0.78-0.97) | 0.72 |
| rs11649743 | 0.83 (0.66-1.05) | 0.88 (0.84-0.93) | 0.44 | 0.87 (0.80-0.94) | 0.88 (0.81-0.94) | 0.86 (0.75-0.99) | 0.99 |
| rs4430796 | 0.81 (0.67-0.97) | 0.80 (0.76-0.83) | 0.96 | 0.80 (0.74-0.85) | 0.81 (0.76-0.85) | 0.78 (0.70-0.88) | 0.95 |
| rs7501939 | 0.81 (0.67-0.98) | 0.83 (0.79-0.86) | 0.70 | 0.82 (0.77-0.88) | 0.82 (0.78-0.88) | 0.84 (0.75-0.94) | 0.76 |
| rs1859962 | 1.16 (0.96-1.39) | 1.19 (1.15-1.24) | 0.83 | 1.25 (1.17-1.33) | 1.16 (1.10-1.23) | 1.10 (0.99-1.23) | 0.03 |
| rs266849 | 0.87 (0.68-1.11) | 0.93 (0.88-0.98) | 0.43 | 0.97 (0.90-1.06) | 0.93 (0.86-1.00) | 0.87 (0.75-1.00) | 0.18 |
| rs2735839 | 0.83 (0.63-1.08) | 0.87 (0.82-0.93) | 0.74 | 0.87 (0.79-0.96) | 0.90 (0.83-0.98) | 0.82 (0.70-0.96) | 0.73 |
| rs5759167 | 0.93 (0.77-1.12) | 0.86 (0.83-0.90) | 0.53 | 0.85 (0.80-0.91) | 0.87 (0.82-0.92) | 0.89 (0.80-0.99) | 0.50 |
| rs5945572 | 1.19 (0.90-1.58) | 1.24 (1.17-1.32) | 0.51 | 1.17 (1.06-1.29) | 1.23 (1.13-1.34) | 1.32 (1.12-1.56) | 0.21 |
| rs5945619 | 1.05 (0.80-1.38) | 1.26 (1.19-1.34) | 0.11 | 1.13 (1.03-1.24) | 1.28 (1.17-1.39) | 1.37 (1.16-1.60) | 0.02 |

^1^ The p-values correspond to a one-degree of freedom likelihood ratio test of the interaction term as implemented in a logistic regression. FH -: no family history of prostate cancer; FH+: family history of prostate cancer
