# Supplementary material for: Characterizing Associations and SNP-Environment Interactions for GWAS-Identified Prostate Cancer Risk Markers—Results from BPC3
Source: PLoS One. 2011 Feb 24;6(2):e17142. doi: 10.1371/journal.pone.0017142 (PMC3044744; doi:10.1371/journal.pone.0017142)
Supplement: Table S5 — Association between height and prostate cancer risk stratified by SNP genotypes. (DOC) [file pone.0017142.s006.doc]

**Supplementary Table 5:** Association between height and prostate cancer risk stratified by SNP genotypes

|  | Height* OR (95% CI) | | |  |  |
| --- | --- | --- | --- | --- | --- |
| SNP | Common allele homozygotes | Heterozygotes | Rare allele homozygotes | P^1^ (Interaction) | P^2^ (joint) |
| rs721048 | 1.05 (0.97-1.14) | 1.01 (0.89-1.14) | 1.06 (0.73-1.53) | 0.45 | 0.28 |
|  | 1.05 (0.96-1.15) | 1.00 (0.87-1.14) | 1.47 (0.98-2.19) |  |  |
| rs2660753 | 1.01 (0.94-1.09) | 1.06 (0.91-1.23) | 1.10 (0.62-1.94) | 0.74 | 0.50 |
|  | 1.05 (0.97-1.14) | 0.98 (0.83-1.15) | 1.10 (0.59-2.05) |  |  |
| rs9364554 | 1.04 (0.94-1.14) | 1.07 (0.96-1.19) | 0.75 (0.60-0.95) | 0.24 | 0.29 |
|  | 1.06 (0.96-1.17) | 1.04 (0.93-1.17) | 0.84 (0.66-1.08) |  |  |
| rs10486567 | 1.05 (0.97-1.15) | 0.99 (0.89-1.11) | 1.08 (0.80-1.45) | 0.90 | 0.42 |
|  | 1.02 (0.93-1.12) | 1.10 (0.97-1.24) | 0.98 (0.72-1.34) |  |  |
| rs6465657 | 1.01 (0.89-1.15) | 1.08 (0.98-1.18) | 0.92 (0.80-1.06) | 0.37 | 0.31 |
|  | 1.05 (0.92-1.21) | 1.05 (0.95-1.17) | 1.00 (0.85-1.16) |  |  |
| rs1016343 | 1.07 (0.97-1.16) | 0.99 (0.88-1.12) | 0.67 (0.49-0.93) | 0.26 | 0.37 |
|  | 1.05 (0.96-1.15) | 1.04 (0.92-1.18) | 0.72 (0.51-1.03) |  |  |
| rs7841060 | 1.05 (0.96-1.14) | 1.02 (0.90-1.14) | 0.65 (0.47-0.90) | 0.56 | 0.54 |
|  | 1.04 (0.94-1.14) | 1.06 (0.94-1.21) | 0.77 (0.55-1.10) |  |  |
| rs16901979 | 1.01 (0.93-1.08) | 0.95 (0.73-1.25) | 0.40 (0.02-6.47) | 0.19 | 0.41 |
|  | 1.02 (0.95-1.11) | 0.85 (0.64-1.13) | 0.10 (0.00-3.94) |  |  |
| rs620861 | 1.07 (0.96-1.20) | 1.05 (0.95-1.17) | 0.78 (0.64-0.95) | 0.53 | 0.55 |
|  | 1.03 (0.92-1.16) | 1.05 (0.94-1.18) | 0.93 (0.76-1.15) |  |  |
| rs6983267 | 0.97 (0.86-1.10) | 1.05 (0.96-1.16) | 1.03 (0.89-1.20) | 0.53 | 0.35 |
|  | 0.97 (0.85-1.12) | 1.08 (0.97-1.19) | 1.07 (0.92-1.25) |  |  |
| rs1447295 | 1.04 (0.96-1.12) | 0.92 (0.79-1.07) | 1.61 (0.87-2.98) | 0.98 | 0.84 |
|  | 1.03 (0.95-1.12) | 0.99 (0.84-1.17) | 0.83 (0.44-1.56) |  |  |
| rs4242382 | 1.04 (0.97-1.13) | 0.93 (0.81-1.08) | 1.71 (0.94-3.09) | 0.63 | 0.46 |
|  | 1.04 (0.96-1.13) | 1.02 (0.87-1.20) | 0.99 (0.54-1.84) |  |  |
| rs7837688 | 1.03 (0.95-1.12) | 0.92 (0.79-1.07) | 1.77 (0.97-3.25) | 0.47 | 0.53 |
|  | 1.03 (0.95-1.12) | 1.00 (0.84-1.17) | 1.26 (0.67-2.38) |  |  |
| rs1571801 | 1.06 (0.97-1.16) | 0.98 (0.87-1.09) | 0.88 (0.67-1.14) | 0.24 | 0.27 |
|  | 1.08 (0.98-1.19) | 1.00 (0.88-1.12) | 0.88 (0.67-1.17) |  |  |
| rs10993994 | 0.99 (0.88-1.11) | 1.08 (0.98-1.20) | 0.96 (0.82-1.14) | 0.73 | 0.33 |
|  | 1.03 (0.91-1.17) | 1.06 (0.96-1.18) | 1.03 (0.86-1.23) |  |  |
| rs12418451 | 1.04 (0.94-1.15) | 0.96 (0.86-1.06) | 1.30 (1.03-1.65) | 0.23 | 0.31 |
|  | 0.97 (0.87-1.08) | 1.08 (0.97-1.21) | 1.14 (0.89-1.46) |  |  |
| rs7931342 | 1.02 (0.89-1.16) | 0.96 (0.87-1.06) | 1.12 (0.97-1.30) | 0.02 | 0.04 |
|  | 0.97 (0.84-1.11) | 1.01 (0.91-1.12) | 1.17 (1.00-1.36) |  |  |
| rs10896449 | 1.02 (0.89-1.15) | 0.98 (0.89-1.08) | 1.16 (1.01-1.33) | 0.02 | 0.03 |
|  | 0.95 (0.83-1.09) | 1.02 (0.92-1.13) | 1.18 (1.01-1.37) |  |  |
| rs11649743 | 1.06 (0.97-1.15) | 0.98 (0.87-1.12) | 0.87 (0.60-1.26) | 0.43 | 0.32 |
|  | 1.06 (0.97-1.16) | 1.03 (0.90-1.18) | 0.73 (0.50-1.08) |  |  |
| rs4430796 | 1.04 (0.91-1.18) | 0.99 (0.89-1.09) | 1.04 (0.90-1.21) | 0.22 | 0.35 |
|  | 0.99 (0.86-1.13) | 1.00 (0.90-1.12) | 1.11 (0.95-1.30) |  |  |
| rs7501939 | 1.08 (0.97-1.21) | 0.95 (0.86-1.06) | 1.06 (0.88-1.28) | 0.44 | 0.48 |
|  | 1.04 (0.93-1.17) | 0.98 (0.88-1.09) | 1.16 (0.96-1.41) |  |  |
| rs1859962 | 1.02 (0.89-1.17) | 1.11 (1.00-1.22) | 0.91 (0.80-1.04) | 0.81 | 0.43 |
|  | 1.09 (0.94-1.26) | 1.04 (0.94-1.16) | 1.01 (0.88-1.17) |  |  |
| rs266849 | 1.04 (0.95-1.13) | 1.02 (0.90-1.15) | 1.15 (0.80-1.65) | 0.72 | 0.27 |
|  | 1.04 (0.96-1.14) | 1.08 (0.94-1.23) | 1.04 (0.70-1.54) |  |  |
| rs2735839 | 1.00 (0.93-1.08) | 1.04 (0.90-1.19) | 1.59 (0.94-2.69) | 0.46 | 0.38 |
|  | 1.05 (0.97-1.15) | 0.99 (0.85-1.15) | 1.12 (0.64-1.97) |  |  |
| rs5945572 | 1.08 (0.99-1.18) |  | 0.91 (0.81-1.02) | 0.40 | 0.51 |
|  | 1.06 (0.96-1.17) |  | 0.98 (0.86-1.10) |  |  |
| rs5945619 | 1.10 (1.01-1.20) |  | 0.91 (0.82-1.02) | 0.52 | 0.47 |
|  | 1.06 (0.97-1.17) |  | 0.99 (0.88-1.11) |  |  |
| rs7127900 | 0.99 (0.91-1.08) | 1.07 (0.95-1.20) | 1.15 (0.82-1.62) | 0.48 | 0.55 |
|  | 1.02 (0.93-1.12) | 1.05 (0.93-1.20) | 0.92 (0.64-1.32) |  |  |
| rs7679673 | 0.99 (0.88-1.11) | 1.05 (0.95-1.16) | 1.14 (0.81-1.61) | 0.71 | 0.77 |
|  | 1.00 (0.89-1.14) | 1.00 (0.90-1.11) | 0.93 (0.64-1.33) |  |  |
| rs16902094 | 0.99 (0.90-1.08) | 1.04 (0.90-1.19) | 1.10 (0.77-1.58) | 0.89 | 0.98 |
|  | 0.97 (0.89-1.07) | 1.06 (0.91-1.23) | 0.95 (0.65-1.40) |  |  |
| rs5759167 | 1.13 (0.99-1.29) | 0.99 (0.90-1.09) | 1.15 (0.82-1.61) | 0.78 | 0.69 |
|  | 1.03 (0.90-1.19) | 1.04 (0.94-1.16) | 0.93 (0.65-1.34) |  |  |
| rs17021918 | 1.00 (0.90-1.11) | 0.97 (0.87-1.07) | 1.11 (0.79-1.57) | 0.31 | 0.45 |
|  | 1.04 (0.93-1.16) | 0.94 (0.84-1.05) | 0.93 (0.65-1.35) |  |  |
| rs1512268 | 0.96 (0.84-1.09) | 1.05 (0.95-1.16) | 1.12 (0.80-1.57) | 0.25 | 0.41 |
|  | 1.06 (0.93-1.22) | 1.01 (0.91-1.13) | 0.90 (0.63-1.30) |  |  |
| rs1465618 | 1.05 (0.96-1.15) | 0.99 (0.88-1.11) | 1.12 (0.80-1.58) | 0.14 | 0.25 |
|  | 1.09 (0.99-1.19) | 0.93 (0.82-1.06) | 0.92 (0.64-1.32) |  |  |
| rs12621278 | 1.02 (0.95-1.09) | 1.00 (0.80-1.25) | 1.13 (0.80-1.58) | 0.79 | 0.74 |
|  | 1.02 (0.95-1.11) | 1.01 (0.79-1.29) | 0.93 (0.65-1.34) |  |  |
| rs12500426 | 1.09 (0.95-1.24) | 1.01 (0.91-1.11) | 1.16 (0.83-1.63) | 0.53 | 0.61 |
|  | 1.05 (0.91-1.20) | 1.04 (0.93-1.15) | 0.97 (0.67-1.39) |  |  |
| rs2928679 | 1.10 (0.97-1.24) | 1.04 (0.94-1.15) | 1.12 (0.80-1.58) | 0.86 | 0.83 |
|  | 0.98 (0.85-1.12) | 1.04 (0.94-1.16) | 0.89 (0.62-1.29) |  |  |

* 173-180 cm and >180 cm respectively, versus <173 cm.

^1^ The Interaction test corresponds to a one-degree of freedom likelihood ratio test of the interaction term as implemented in a logistic regression.

^2^ The Joint test corresponds to a two-degree of freedom likelihood ratio test of the interaction term and the environmental main effect as implemented in a logistic regression
