# Supplementary material for: Characterizing Associations and SNP-Environment Interactions for GWAS-Identified Prostate Cancer Risk Markers—Results from BPC3
Source: PLoS One. 2011 Feb 24;6(2):e17142. doi: 10.1371/journal.pone.0017142 (PMC3044744; doi:10.1371/journal.pone.0017142)
Supplement: Table S6 — Association between smoking and prostate cancer risk stratified by SNP genotypes. (DOC) [file pone.0017142.s007.doc]

**Supplementary Table 6:** Association between smoking and prostate cancer risk stratified by SNP genotypes

|  | Smoking Status* OR (95% CI) | | |  |  |
| --- | --- | --- | --- | --- | --- |
| SNP | Common allele homozygotes | Heterozygotes | Rare allele homozygotes | P^1^ (Interaction) | P^2^ (joint) |
| rs721048 | 0.94 (0.88-1.02) | 0.97 (0.87-1.09) | 0.99 (0.71-1.39) | 0.87 | 0.15 |
|  | 0.90 (0.79-1.03) | 0.90 (0.75-1.08) | 1.16 (0.64-2.08) |  |  |
| rs1465618 | 0.99 (0.91-1.07) | 0.94 (0.84-1.05) | 1.14 (0.82-1.58) | 0.27 | 0.21 |
|  | 0.93 (0.82-1.07) | 0.94 (0.78-1.13) | 1.54 (0.88-2.68) |  |  |
| rs12621278 | 0.96 (0.89-1.02) | 0.98 (0.80-1.20) | 1.11 (0.80-1.53) | 0.73 | 0.34 |
|  | 0.95 (0.85-1.07) | 0.80 (0.57-1.14) | 1.54 (0.88-2.69) |  |  |
| rs2660753 | 0.94 (0.88-1.01) | 0.99 (0.86-1.13) | 0.97 (0.57-1.64) | 0.29 | 0.08 |
|  | 0.95 (0.84-1.07) | 0.74 (0.58-0.94) | 1.22 (0.54-2.74) |  |  |
| rs17021918 | 1.01 (0.92-1.11) | 0.97 (0.88-1.07) | 1.08 (0.78-1.50) | 0.33 | 0.15 |
|  | 0.90 (0.77-1.05) | 1.04 (0.89-1.23) | 1.50 (0.86-2.61) |  |  |
| rs12500426 | 0.84 (0.75-0.95) | 1.01 (0.93-1.11) | 1.08 (0.78-1.50) | 0.29 | 0.14 |
|  | 0.84 (0.69-1.03) | 0.96 (0.83-1.12) | 1.49 (0.85-2.61) |  |  |
| rs7679673 | 0.89 (0.80-0.99) | 0.99 (0.90-1.09) | 1.07 (0.77-1.48) | 0.37 | 0.21 |
|  | 0.96 (0.80-1.14) | 0.92 (0.79-1.08) | 1.57 (0.89-2.76) |  |  |
| rs9364554 | 1.00 (0.92-1.10) | 0.90 (0.82-0.99) | 0.90 (0.73-1.12) | 0.46 | 0.10 |
|  | 0.92 (0.80-1.07) | 0.92 (0.78-1.08) | 0.78 (0.54-1.13) |  |  |
| rs10486567 | 0.98 (0.90-1.06) | 0.86 (0.77-0.95) | 0.96 (0.73-1.27) | 0.30 | 0.04 |
|  | 0.93 (0.81-1.06) | 0.89 (0.75-1.06) | 0.77 (0.47-1.26) |  |  |
| rs6465657 | 0.84 (0.75-0.95) | 1.05 (0.96-1.15) | 0.88 (0.77-1.00) | 0.51 | 0.09 |
|  | 0.82 (0.67-0.99) | 0.95 (0.82-1.10) | 0.91 (0.73-1.14) |  |  |
| rs1512268 | 0.97 (0.86-1.09) | 0.98 (0.90-1.07) | 1.08 (0.78-1.50) | 0.36 | 0.19 |
|  | 1.09 (0.90-1.32) | 0.92 (0.79-1.08) | 1.52 (0.87-2.66) |  |  |
| rs2928679 | 1.03 (0.91-1.15) | 0.93 (0.85-1.01) | 1.11 (0.81-1.54) | 0.35 | 0.30 |
|  | 0.98 (0.80-1.19) | 0.94 (0.81-1.10) | 1.60 (0.92-2.78) |  |  |
| rs1016343 | 1.00 (0.92-1.08) | 0.92 (0.82-1.02) | 0.95 (0.71-1.27) | 0.64 | 0.31 |
|  | 0.93 (0.81-1.07) | 0.89 (0.74-1.08) | 1.04 (0.63-1.73) |  |  |
| rs7841060 | 0.99 (0.92-1.08) | 0.92 (0.82-1.02) | 0.90 (0.67-1.21) | 0.27 | 0.14 |
|  | 0.93 (0.81-1.07) | 0.87 (0.72-1.04) | 1.05 (0.64-1.72) |  |  |
| rs16901979 | 0.99 (0.92-1.06) | 0.78 (0.61-1.00) | 0.93 (0.11-8.18) | 0.47 | 0.41 |
|  | 0.94 (0.83-1.05) | 0.95 (0.63-1.44) | 5.77 (0.24-136.77) |  |  |
| rs620861 | 0.92 (0.83-1.02) | 1.04 (0.94-1.14) | 0.88 (0.74-1.05) | 0.54 | 0.34 |
|  | 0.90 (0.76-1.06) | 1.00 (0.85-1.17) | 0.84 (0.62-1.15) |  |  |
| rs6983267 | 0.97 (0.86-1.09) | 0.92 (0.84-1.01) | 0.98 (0.86-1.12) | 0.58 | 0.12 |
|  | 0.98 (0.81-1.18) | 0.89 (0.77-1.03) | 0.88 (0.69-1.11) |  |  |
| rs1447295 | 0.97 (0.90-1.04) | 0.94 (0.81-1.09) | 0.97 (0.53-1.76) | 0.70 | 0.30 |
|  | 0.91 (0.80-1.02) | 0.97 (0.76-1.23) | 2.06 (0.71-5.93) |  |  |
| rs4242382 | 0.95 (0.89-1.02) | 0.89 (0.77-1.02) | 1.10 (0.63-1.95) | 0.92 | 0.13 |
|  | 0.89 (0.79-1.00) | 0.97 (0.77-1.22) | 3.83 (1.04-14.15) |  |  |
| rs7837688 | 0.96 (0.90-1.03) | 0.95 (0.82-1.10) | 0.95 (0.52-1.72) | 0.32 | 0.25 |
|  | 0.92 (0.82-1.04) | 0.97 (0.76-1.23) | 1.70 (0.59-4.89) |  |  |
| rs16902094 | 0.94 (0.87-1.02) | 1.04 (0.91-1.18) | 1.02 (0.72-1.44) | 0.07 | 0.06 |
|  | 0.96 (0.84-1.10) | 0.96 (0.77-1.19) | 1.23 (0.68-2.22) |  |  |
| rs1571801 | 0.94 (0.86-1.02) | 0.95 (0.86-1.05) | 1.10 (0.86-1.41) | 0.87 | 0.19 |
|  | 0.89 (0.78-1.03) | 0.94 (0.79-1.12) | 0.94 (0.62-1.43) |  |  |
| rs10993994 | 0.94 (0.85-1.05) | 0.97 (0.89-1.06) | 0.90 (0.78-1.05) | 0.15 | 0.07 |
|  | 0.99 (0.83-1.18) | 0.92 (0.79-1.07) | 0.84 (0.66-1.08) |  |  |
| rs7127900 | 0.97 (0.90-1.05) | 0.92 (0.82-1.02) | 1.11 (0.80-1.53) | 0.51 | 0.33 |
|  | 0.90 (0.79-1.04) | 0.92 (0.76-1.10) | 1.63 (0.94-2.83) |  |  |
| rs12418451 | 0.96 (0.87-1.05) | 0.93 (0.84-1.02) | 1.11 (0.89-1.38) | 0.18 | 0.09 |
|  | 1.05 (0.90-1.22) | 0.83 (0.71-0.98) | 0.71 (0.49-1.01) |  |  |
| rs7931342 | 1.02 (0.91-1.15) | 0.90 (0.82-0.98) | 1.02 (0.89-1.17) | 0.96 | 0.27 |
|  | 0.76 (0.63-0.93) | 0.97 (0.83-1.12) | 1.05 (0.84-1.31) |  |  |
| rs10896449 | 0.98 (0.87-1.10) | 0.89 (0.81-0.97) | 1.01 (0.89-1.15) | 0.66 | 0.09 |
|  | 0.76 (0.62-0.93) | 0.93 (0.80-1.08) | 1.06 (0.86-1.32) |  |  |
| rs11649743 | 0.97 (0.90-1.05) | 0.88 (0.79-0.99) | 1.02 (0.74-1.42) | 0.27 | 0.06 |
|  | 0.91 (0.80-1.04) | 0.89 (0.73-1.07) | 1.04 (0.59-1.83) |  |  |
| rs4430796 | 1.01 (0.90-1.14) | 0.89 (0.82-0.98) | 0.95 (0.83-1.09) | 0.03 | 0.01 |
|  | 1.12 (0.92-1.38) | 0.82 (0.71-0.95) | 0.88 (0.70-1.10) |  |  |
| rs7501939 | 1.05 (0.94-1.16) | 0.90 (0.82-0.99) | 0.95 (0.81-1.12) | 0.01 | 0.01 |
|  | 1.14 (0.96-1.36) | 0.85 (0.73-0.99) | 0.87 (0.65-1.15) |  |  |
| rs1859962 | 0.94 (0.83-1.07) | 0.97 (0.88-1.05) | 0.93 (0.82-1.05) | 0.48 | 0.13 |
|  | 0.79 (0.64-0.97) | 1.04 (0.89-1.20) | 0.85 (0.69-1.05) |  |  |
| rs266849 | 0.94 (0.87-1.02) | 0.98 (0.87-1.09) | 0.90 (0.65-1.24) | 0.59 | 0.12 |
|  | 0.89 (0.78-1.01) | 0.92 (0.76-1.11) | 1.42 (0.80-2.52) |  |  |
| rs2735839 | 0.97 (0.90-1.04) | 0.93 (0.82-1.06) | 0.66 (0.42-1.05) | 0.74 | 0.21 |
|  | 0.90 (0.79-1.01) | 1.07 (0.86-1.32) | 0.37 (0.15-0.91) |  |  |
| rs5759167 | 0.94 (0.83-1.06) | 0.98 (0.89-1.07) | 1.11 (0.80-1.54) | 0.4 | 0.24 |
|  | 0.84 (0.68-1.02) | 0.99 (0.85-1.15) | 1.58 (0.91-2.76) |  |  |
| rs5945572 | 0.97 (0.90-1.06) |  | 0.96 (0.86-1.07) | 0.46 | 0.30 |
|  | 0.88 (0.77-1.01) |  | 1.02 (0.85-1.21) |  |  |
| rs5945619 | 0.96 (0.89-1.04) |  | 0.95 (0.85-1.05) | 0.32 | 0.09 |
|  | 0.86 (0.75-0.98) |  | 0.99 (0.84-1.18) |  |  |

* Current and former smokers respectively versus never smokers.

^1^ The Interaction test corresponds to a one-degree of freedom likelihood ratio test of the interaction term as implemented in a logistic regression.

^2^ The Joint test corresponds to a two-degree of freedom likelihood ratio test of the interaction term and the environmental main effect as implemented in a logistic regression.
