# Supplementary material for: Characterizing Associations and SNP-Environment Interactions for GWAS-Identified Prostate Cancer Risk Markers—Results from BPC3
Source: PLoS One. 2011 Feb 24;6(2):e17142. doi: 10.1371/journal.pone.0017142 (PMC3044744; doi:10.1371/journal.pone.0017142)
Supplement: Table S7 — Association between alcohol consumption and prostate cancer risk stratified by SNP genotypes. (DOC) [file pone.0017142.s008.doc]

**Supplementary Table 7:** Association between alcohol consumption and prostate cancer risk stratified by SNP genotypes

.

|  | Alcohol consumption* OR (95% CI) | | |  |  |
| --- | --- | --- | --- | --- | --- |
| SNP | Common Homozygotes | Heterozygotes | Rare Homozygotes | P^1^ (Interaction) | P^2^ (joint) |
| rs721048 | 1.11 (1.01-1.22) | 1.05 (0.91-1.21) | 1.11 (0.73-1.68) | 0.47 | 0.08 |
| rs1465618 | 1.11 (1.00-1.23) | 1.09 (0.95-1.25) | 1.02 (0.67-1.58) | 0.48 | 0.10 |
| rs12621278 | 1.09 (1.00-1.18) | 1.15 (0.87-1.52) | 1.08 (0.70-1.67) | 0.74 | 0.12 |
| rs2660753 | 1.12 (1.02-1.22) | 1.03 (0.86-1.24) | 0.80 (0.39-1.64) | 0.24 | 0.04 |
| rs17021918 | 1.18 (1.04-1.33) | 1.03 (0.91-1.16) | 1.05 (0.68-1.62) | 0.04 | 0.02 |
| rs12500426 | 1.04 (0.89-1.21) | 1.08 (0.96-1.21) | 1.07 (0.69-1.65) | 0.14 | 0.03 |
| rs7679673 | 1.26 (1.10-1.45) | 1.02 (0.90-1.14) | 0.95 (0.61-1.46) | 0.03 | 0.01 |
| rs9364554 | 1.08 (0.97-1.20) | 1.13 (1.00-1.29) | 0.97 (0.74-1.29) | 0.97 | 0.11 |
| rs10486567 | 1.06 (0.96-1.17) | 1.11 (0.97-1.26) | 1.05 (0.73-1.51) | 0.98 | 0.20 |
| rs6465657 | 1.15 (0.98-1.34) | 1.01 (0.90-1.13) | 1.21 (1.02-1.43) | 0.62 | 0.11 |
| rs1512268 | 1.11 (0.95-1.28) | 1.07 (0.95-1.20) | 1.02 (0.66-1.57) | 0.70 | 0.10 |
| rs2928679 | 1.14 (0.99-1.32) | 1.04 (0.92-1.17) | 1.06 (0.69-1.63) | 0.91 | 0.13 |
| rs1016343 | 1.10 (0.99-1.22) | 1.07 (0.93-1.23) | 1.48 (1.01-2.17) | 0.28 | 0.03 |
| rs7841060 | 1.10 (0.99-1.22) | 1.06 (0.92-1.22) | 1.45 (0.98-2.14) | 0.38 | 0.05 |
| rs16901979 | 1.10 (1.01-1.21) | 1.20 (0.85-1.68) | NA | 0.44 | 0.04 |
| rs620861 | 1.03 (0.90-1.17) | 1.22 (1.07-1.38) | 0.94 (0.73-1.19) | 0.93 | 0.09 |
| rs6983267 | 1.03 (0.89-1.20) | 1.13 (1.01-1.27) | 1.02 (0.86-1.21) | 0.85 | 0.15 |
| rs1447295 | 1.14 (1.04-1.25) | 0.99 (0.82-1.18) | 1.04 (0.49-2.22) | 0.12 | 0.02 |
| rs4242382 | 1.15 (1.05-1.26) | 0.92 (0.78-1.09) | 0.98 (0.49-1.97) | 0.02 | 0.004 |
| rs7837688 | 1.15 (1.05-1.26) | 0.94 (0.79-1.13) | 1.05 (0.50-2.21) | 0.05 | 0.01 |
| rs16902094 | 1.06 (0.96-1.19) | 1.17 (0.99-1.39) | 0.98 (0.61-1.59) | 0.37 | 0.12 |
| rs1571801 | 1.09 (0.97-1.21) | 1.13 (0.99-1.29) | 1.08 (0.79-1.49) | 0.57 | 0.05 |
| rs10993994 | 1.02 (0.89-1.17) | 1.11 (0.99-1.25) | 1.18 (0.97-1.43) | 0.36 | 0.08 |
| rs7127900 | 1.18 (1.07-1.31) | 0.96 (0.83-1.11) | 1.03 (0.67-1.58) | 0.06 | 0.02 |
| rs12418451 | 1.14 (1.02-1.29) | 1.10 (0.97-1.25) | 0.90 (0.69-1.17) | 0.24 | 0.04 |
| rs7931342 | 1.07 (0.92-1.25) | 1.07 (0.95-1.20) | 1.11 (0.93-1.31) | 0.86 | 0.19 |
| rs10896449 | 1.08 (0.93-1.25) | 1.07 (0.95-1.20) | 1.12 (0.95-1.32) | 0.89 | 0.14 |
| rs11649743 | 1.02 (0.93-1.13) | 1.23 (1.07-1.42) | 1.06 (0.67-1.68) | 0.18 | 0.05 |
| rs4430796 | 1.01 (0.87-1.18) | 1.12 (1.00-1.26) | 1.13 (0.95-1.34) | 0.40 | 0.09 |
| rs7501939 | 1.10 (0.96-1.26) | 1.09 (0.97-1.22) | 1.21 (0.97-1.51) | 0.80 | 0.06 |
| rs1859962 | 1.05 (0.90-1.23) | 1.16 (1.03-1.30) | 1.03 (0.88-1.20) | 0.63 | 0.08 |
| rs266849 | 1.10 (1.00-1.21) | 1.07 (0.92-1.24) | 1.05 (0.66-1.67) | 0.69 | 0.12 |
| rs2735839 | 1.10 (1.00-1.21) | 1.03 (0.87-1.22) | 0.90 (0.49-1.65) | 0.34 | 0.11 |
| rs5759167 | 1.10 (0.94-1.28) | 1.16 (1.03-1.30) | 1.03 (0.67-1.59) | 0.26 | 0.04 |
| rs5945572 | 1.07 (0.96-1.18) |  | 1.16 (1.01-1.33) | 0.36 | 0.06 |
| rs5945619 | 1.04 (0.94-1.15) |  | 1.18 (1.04-1.35) | 0.15 | 0.04 |

^*^ ≥30 vs. <30 g ethanol

^1^ The Interaction test corresponds to a one-degree of freedom likelihood ratio test of the interaction term as implemented in a logistic regression.

^2^ The Joint test corresponds to a two-degree of freedom likelihood ratio test of the interaction term and the environmental main effect as implemented in a logistic regression.
