# Supplementary material for: Characterizing Associations and SNP-Environment Interactions for GWAS-Identified Prostate Cancer Risk Markers—Results from BPC3
Source: PLoS One. 2011 Feb 24;6(2):e17142. doi: 10.1371/journal.pone.0017142 (PMC3044744; doi:10.1371/journal.pone.0017142)
Supplement: Table S8 — Pair-wise SNP-SNP interactions that reached a p-value < 0.05. (DOC) [file pone.0017142.s009.doc]

**Supplementary Table 8:** Pair-wise SNP-SNP interactions that reached a p-value < 0.05

| SNP1 | SNP2 | Interaction OR (95% CI) | P |
| --- | --- | --- | --- |
| rs620861 | rs5945619 | 1.17 (1.07-1.28) | 0.0005 |
| rs620861 | rs5945572 | 1.16 (1.06-1.26) | 0.0013 |
| rs11649743 | rs16902094 | 0.84 (0.76-0.94) | 0.0022 |
| rs2660753 | rs9364554 | 0.87 (0.79-0.96) | 0.0059 |
| rs5945572 | rs7679673 | 1.13 (1.03-1.23) | 0.0069 |
| rs12418451 | rs1465618 | 1.11 (1.03-1.20) | 0.0071 |
| rs1016343 | rs16901979 | 1.32 (1.07-1.63) | 0.0090 |
| rs620861 | rs2735839 | 1.12 (1.03-1.23) | 0.011 |
| rs1016343 | rs5759167 | 0.91 (0.85-0.98) | 0.011 |
| rs6465657 | rs7679673 | 0.93 (0.87-0.98) | 0.013 |
| rs620861 | rs11649743 | 1.10 (1.02-1.19) | 0.014 |
| rs620861 | rs4242382 | 0.89 (0.81-0.98) | 0.015 |
| rs16901979 | rs12621278 | 1.57 (1.08-2.28) | 0.016 |
| rs4430796 | rs2928679 | 1.07 (1.01-1.14) | 0.018 |
| rs5945619 | rs7679673 | 1.11 (1.02-1.21) | 0.019 |
| rs4242382 | rs12500426 | 0.90 (0.82-0.98) | 0.020 |
| rs6983267 | rs1512268 | 0.93 (0.88-0.99) | 0.021 |
| rs4242382 | rs17021918 | 1.12 (1.02-1.23) | 0.022 |
| rs2660753 | rs2735839 | 1.16 (1.02-1.31) | 0.022 |
| rs7841060 | rs5759167 | 0.92 (0.86-0.99) | 0.022 |
| rs4242382 | rs7931342 | 0.90 (0.83-0.99) | 0.024 |
| rs7837688 | rs17021918 | 1.12 (1.01-1.23) | 0.025 |
| rs6983267 | rs10993994 | 1.07 (1.01-1.13) | 0.026 |
| rs7127900 | rs1465618 | 1.10 (1.01-1.20) | 0.026 |
| rs1465618 | rs12500426 | 1.08 (1.01-1.16) | 0.027 |
| rs4242382 | rs10896449 | 0.91 (0.83-0.99) | 0.027 |
| rs4430796 | rs12621278 | 0.86 (0.75-0.98) | 0.028 |
| rs1859962 | rs12621278 | 0.87 (0.76-0.98) | 0.028 |
| rs10486567 | rs7679673 | 1.08 (1.01-1.16) | 0.028 |
| rs10993994 | rs2735839 | 1.10 (1.01-1.19) | 0.030 |
| rs1447295 | rs17021918 | 1.11 (1.01-1.22) | 0.032 |
| rs721048 | rs7501939 | 1.09 (1.01-1.17) | 0.032 |
| rs620861 | rs1447295 | 0.90 (0.82-0.99) | 0.032 |
| rs7931342 | rs12500426 | 0.94 (0.89-0.99) | 0.033 |
| rs7837688 | rs12500426 | 0.91 (0.83-0.99) | 0.034 |
| rs721048 | rs4430796 | 1.08 (1.01-1.17) | 0.035 |
| rs7501939 | rs16902094 | 1.10 (1.01-1.20) | 0.035 |
| rs1447295 | rs12500426 | 0.91 (0.83-0.99) | 0.036 |
| rs6465657 | rs1465618 | 0.93 (0.87-1.00) | 0.036 |
| rs1571801 | rs12418451 | 0.93 (0.86-1.00) | 0.036 |
| rs1447295 | rs7931342 | 0.91 (0.83-0.99) | 0.037 |
| rs7841060 | rs12500426 | 0.93 (0.86-1.00) | 0.038 |
| rs1512268 | rs12621278 | 1.15 (1.01-1.31) | 0.038 |
| rs10993994 | rs4430796 | 1.06 (1.00-1.13) | 0.040 |
| rs1447295 | rs10896449 | 0.91 (0.83-1.00) | 0.041 |
| rs7841060 | rs16901979 | 1.24 (1.00-1.54) | 0.044 |
| rs7501939 | rs12621278 | 0.87 (0.76-1.00) | 0.044 |
| rs7837688 | rs7679673 | 0.91 (0.83-1.00) | 0.046 |
| rs4242382 | rs16902094 | 1.13 (1.00-1.28) | 0.048 |
| rs6983267 | rs5759167 | 1.06 (1.00-1.12) | 0.049 |
